# Skullcapflavone II Inhibits SLC1A4‐Mediated L‐Serine Uptake and Promotes Mitochondrial Damage in Gastric Cancer

**DOI:** 10.1002/advs.202417225

**Published:** 2025-09-19

**Authors:** Jing Zhao, Yu‐Bo Ma, Rui‐Hong Xia, Zheng‐Chen Jiang, Ying Zhou, Ya‐Nan Wang, Meng‐Yan Yang, Jing‐Jie Dai, Tao Zhu, Li‐Bin Pan, Li Yuan

**Affiliations:** ^1^ Zhejiang Provincial Research Center for Upper Gastrointestinal Tract Cancer Zhejiang Cancer Hospital Hangzhou 310022 China; ^2^ Zhejiang Key Lab of Prevention Diagnosis and Therapy of Upper Gastrointestinal Cancer Zhejiang Cancer Hospital Hangzhou 310022 China; ^3^ School of Life Sciences Tianjin University Tianjin 300072 China; ^4^ Department of Gastric Surgery Zhejiang Cancer Hospital Hangzhou 310022 China; ^5^ Department of Pharmacy Zhejiang Cancer Hospital Hangzhou 310022 China; ^6^ Postgraduate training base Alliance of Wenzhou Medical University (Zhejiang Cancer Hospital) Hangzhou 310022 China

**Keywords:** gastric cancer, L‐serine uptake, serine metabolism, skullcapflavone II, SLC1A4

## Abstract

Gastric cancer (GC) is one of the most lethal human malignancies worldwide. Serine metabolism is essential for meeting biosynthetic demands and regulating the redox state of GC cells. This study demonstrates that Skullcapflavone II (SkII) selectively inhibits the proliferation and metastasis of GC cells. Transcriptomic and metabolomic analyses reveal that SkII treatment significantly affects serine metabolism in GC cells, and isotope tracing experiments confirmed that SkII reduces intracellular L‐serine levels by inhibiting uptake rather than de novo synthesis. Furthermore, IHC analysis reveal significant upregulation of the L‐serine transporter SLC1A4 in GC tissues. Binding studies using PELSA, SPR, DARTS, CETSA, and MD suggest that SLC1A4 is a potential direct target of SkII. SkII‐induced disruption of serine metabolism resulted in an imbalance in GSSG/GSH, leading to increased accumulation of intracellular ROS and oxidative stress. This metabolic disruption causes mitochondrial damage, impaired energy production, and increased apoptosis in GC cells. Additionally, in vivo studies reveal that a serine and glycine deficient diet significantly enhanced the antitumor efficacy of SkII, highlighting its potential as a combinatorial therapeutic strategy. These findings provide compelling evidence that SkII is a novel candidate for targeting serine metabolism, suggesting a promising therapeutic approach for treating GC.

## Graphical abstract

Skullcapflavone II (SkII) significantly alters serine metabolism in gastric cancer cells by directly targeting the L‐serine transporter SLC1A4, thereby inhibiting L‐serine uptake rather than de novo synthesis. This disruption of serine metabolism by SkII leads to increased oxidative stress and consequent mitochondrial damage.

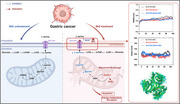

## 1 Introduction

Gastric cancer (GC) is the fifth most commonly diagnosed cancer worldwide, accounting for 6.8% of all cancer‐related deaths in 2022 and ranking as the fifth leading cause of cancer mortality.^[^ ^1^ ^]^ Surgery remains the cornerstone of GC treatment. However, owing to the limitations of early screening technologies, the majority of cases are diagnosed at an advanced stage.^[^ ^2^ ^]^ Despite advancements in surgical techniques, chemotherapy, and targeted therapies, the overall 5‐year survival rate for patients with GC remains below 30%.^[^ ^3^ ^]^ Therefore, the development of innovative pharmacological strategies to treat patients with advanced GC effectively is urgently needed.

Metabolic reprogramming is a key hallmark of cancer progression. Cancer cells often reprogram their metabolic pathways to meet high anabolic demands and maintain redox balance during cancer progression.^[^ ^4^, ^5^ ^]^ Over the past decade, numerous studies have highlighted the importance of a nonessential amino acid, L‐serine, as a critical nutrient that supports cancer cell metabolism, tumor growth, and therapy resistance.^[^ ^6^, ^7^ ^]^ Intracellular L‐serine can be generated through the de novo biosynthesis pathway or acquired via cellular transport, and subsequently utilized for glycine and cysteine biosynthesis as well as antioxidant defense mechanisms.^[^ ^8^ ^]^ The de novo serine biosynthesis pathway converts the glycolytic intermediate 3‐phosphoglycerate (3‐PG) into 3‐phosphoserine (3‐PS) through enzymatic reactions catalyzed by phosphoglycerate dehydrogenase (PHGDH) and phosphoserine aminotransferase 1 (PSAT1), followed by the conversion of 3‐PS to L‐serine by phosphoserine phosphatase (PSPH).^[^ ^9^ ^]^ Several studies have focused on developing PHGDH inhibitors as potential cancer therapies on the basis of the de novo serine biosynthesis pathway.^[^ ^10^, ^11^ ^]^ However, many cancer cells exhibit a limited capacity for de novo serine synthesis, and increased uptake of exogenous L‐serine is often sufficient to compensate for the inhibition of the biosynthetic pathway.^[^ ^12^ ^]^ Preclinical studies have reported that restricting the exogenous supply of L‐serine and glycine to target serine metabolism results in potent anticancer effects.^[^ ^13^ ^]^ Therefore, restricting exogenous L‐serine uptake may confer therapeutic benefits for GC treatment.

Solute carrier (SLC) transporters serve as gatekeepers for the cellular influx and efflux of amino acids, orchestrating a complex network of transport systems to sustain intracellular amino acid homeostasis.^[^ ^14^ ^]^ Notably, several SLC transporters are upregulated in cancer, enabling tumors to acquire diverse nutrients from the extracellular milieu to meet their heightened metabolic demands.^[^ ^15^ ^]^ Consequently, targeting SLC‐mediated nutrient uptake represents a promising therapeutic strategy for cancer treatment. Within the serine metabolic network, multiple members of the SLC family, including SLC1A4, SLC1A5, SLC6A14, and SLC12A4, collectively mediate cellular L‐serine uptake.^[^ ^16^, ^17^ ^]^ Current evidence demonstrates that SLC1A5 serves as the predominant contributor to L‐serine uptake in breast cancer, whereas SLC6A14 plays a major role in colorectal cancer.^[^ ^12^, ^18^ ^]^ SLC1A4 has been identified as the dominant transporter mediating L‐serine uptake in leukemia and at the blood‐brain barrier.^[^ ^19^, ^20^ ^]^ However, the characterization of the principal L‐serine transporter in GC cells remains to be elucidated.

Skullcapflavone II (SkII) is a flavonoid compound derived from *Scutellaria* that has various pharmacological activities, including anti‐inflammatory, hepatoprotective, and neuroprotective effects.^[^ ^21^, ^22^ ^]^ In addition, SkII can regulate the differentiation, survival, and function of osteoclasts.^[^ ^23^ ^]^ Many flavonoid compounds derived from *Scutellaria*, including baicalein, wogonin, and baicalin, have been shown to have anticancer effects by regulating cellular metabolism, modulating ROS‐scavenging enzyme activity, and inhibiting cancer cell proliferation and invasiveness.^[^ ^24^ ^]^ Moreover, SkII significantly inhibited the proliferation, invasion, and migration of breast cancer cells.^[^ ^25^ ^]^ However, systematic research on the role and mechanisms of SkII in GC is lacking.

In this study, we report the anti‐GC effects and underlying mechanisms of SkII. Isotope labeling experiments demonstrated that SkII disrupts serine metabolism in GC cells by inhibiting L‐serine uptake. We provide compelling evidence that the overexpression of the L‐serine transporter SLC1A4 functions as an oncogenic driver in GC. Mechanistically, SLC1A4 serves as a major mediator of L‐serine uptake in GC cell lines. Furthermore, we demonstrate that SkII directly binds to and inhibits SLC1A4 activity, consequently suppressing cellular L‐serine acquisition. SkII treatment also significantly increases intracellular ROS levels, induces mitochondrial damage, affects energy metabolism, and exerts antitumor effects in subcutaneous xenograft, organoids, orthotopic and patient‐derived xenograft models (PDX) of GC. Collectively, our findings provide a potential promising therapeutic strategy for GC.

## 2 Results

### 2.1 SkII Inhibits the Proliferation, Invasion, and Migration of GC Cells In Vitro, While Promoting their Apoptosis

**Figure 1:**
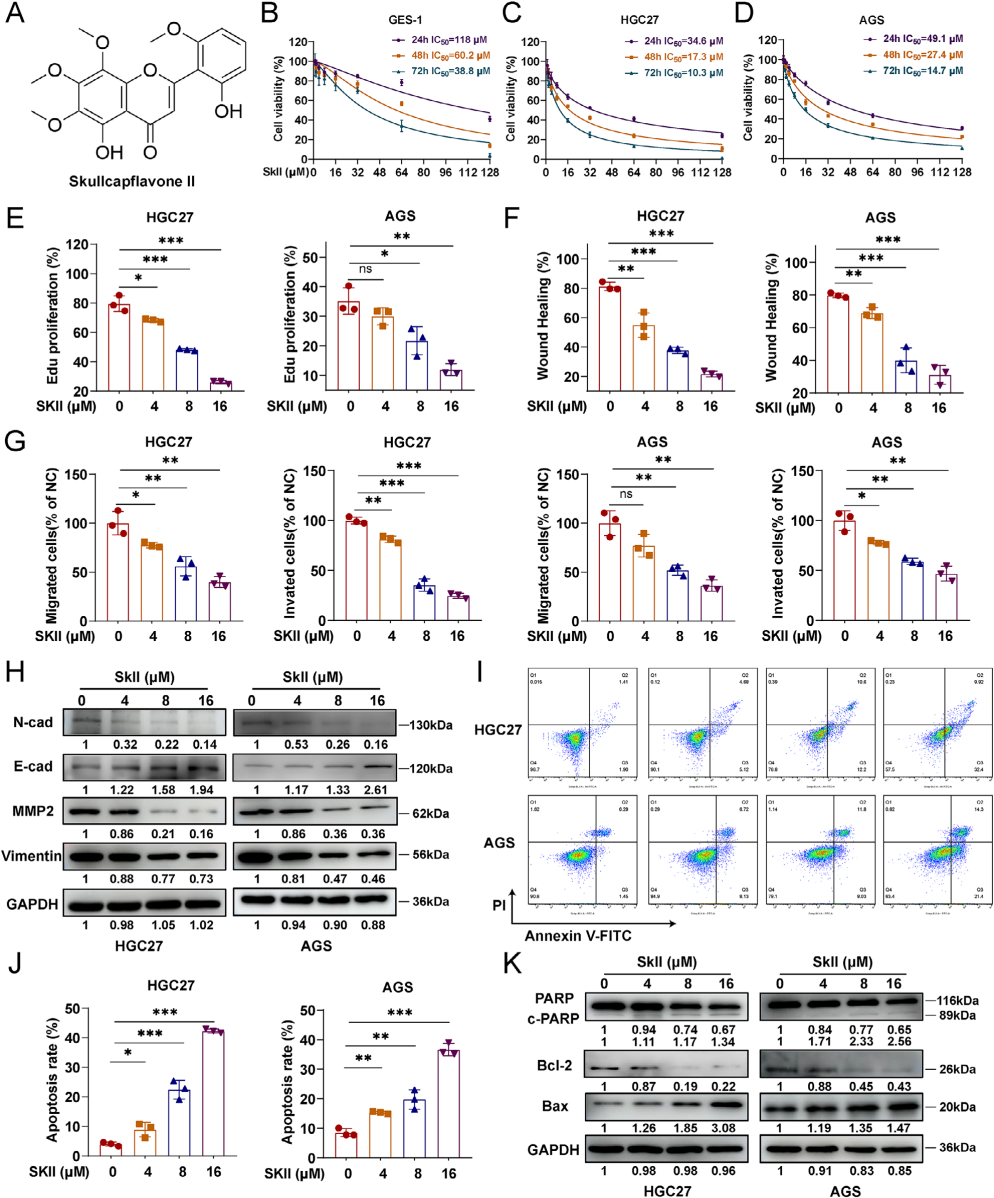
SkII inhibits the proliferation, invasion, and migration and promotes the apoptosis of GC cells. A) Chemical structure of SkII. B‐D) GES‐1, HGC27, and AGS cells were treated with different concentrations of SkII (0–128 µM) for 24, 48, and 72 h. Cell viability was assessed by CCK‐8 assay. E) HGC27 and AGS cells were treated with SkII (0–16 µM) for 48 h and their proliferation ability was assessed using an EdU assay. F) HGC27 and AGS cells were treated with SkII (0–16 µM) for 48 h, and a wound healing assay was used to evaluate their migration capacity. G) Transwell migration and invasion assays of HGC27 and AGS cells treated with SkII (0–16 µM) for 48 h. H) Western blot analysis of N‐cadherin, E‐cadherin, MMP2, Vimentin, and GAPDH (loading control) levels in HGC27 and AGS cells treated with SkII (0–16 µM) for 48 h. I‐J) Flow cytometry analysis of the effect of SkII (0–16 µM) treatment for 48 h on the apoptosis of HGC27 and AGS cells. K) Western blot analysis of cleaved‐PARP, PARP, Bcl‐2, Bax, and GAPDH (loading control) levels in HGC27 and AGS cells treated with SkII (0–16 µM) for 48 h. For all the statistical data, the data are presented as the means ± SDs, and statistical significance was determined by one‐way ANOVA. *Ns*, not significant; *p < 0.05, **p < 0.01, ***p < 0.001.

To evaluate the cytotoxic and inhibitory effects of SkII on GC cells, human normal gastric epithelial cells (GES‐1) and human GC cell lines (HGC27 and AGS) were treated with SkII, and the structural formula of SkII is shown in Figure **1**A. Cytotoxicity assays demonstrated that, with increasing time and concentration, SkII exhibited greater cytotoxicity against the GC cell lines HGC27 and AGS than against the gastric epithelial cell line GES‐1 (Figure 1B–D). The IC_50_ values for GES‐1 cells were 118.0 µM (24 h), 60.2 µM (48 h), and 38.8 µM (72 h), whereas those for HGC27 cells were 34.6 µM (24 h), 17.3 µM (48 h), and 10.3 µM (72 h), and those for AGS cells were 49.1 µM (24 h), 27.4 µM (48 h), and 14.7 µM (72 h). Based on the CCK‐8 results, concentrations of 0, 4, 8, and 16 µM were selected for subsequent experiments. Treatment of HGC27 and AGS cells with SkII led to a dose‐dependent reduction in the proportion of EdU‐positive cells, indicating inhibition of GC cell proliferation, which was further confirmed by colony formation assays (Figure 1E and Figure S1A,B, Supporting Information). We also performed scratch and Transwell assays to assess the effects of SkII on GC cell migration and invasion. Both wound healing area and number of cells that passed through the chamber decreased with increasing concentrations of SkII (Figure 1F,G and Figure S1C,D, Supporting Information). Furthermore, SkII treatment downregulated the expression of mesenchymal markers associated with the EMT pathway, such as N‐cadherin, MMP2, and Vimentin, while SkII treatment upregulated the epithelial marker E‐cadherin (Figure 1H). In addition, we investigated whether SkII triggered apoptosis in GC cells. Flow cytometry analysis with Annexin V‐FITC/PI double staining revealed that SkII induced apoptosis in HGC27 and AGS cells in a dose‐dependent manner (Figure 1I,J). The Western blot results revealed a dose‐dependent increase in the expression of proapoptotic proteins (cleaved PARP and Bax), whereas the expression of the antiapoptotic protein Bcl‐2 was decreased (Figure 1K). Collectively, these findings suggest that SkII effectively inhibits GC cell proliferation, invasion, and migration, and promotes apoptosis, providing a basis for the use of SkII as a potential therapeutic for GC.

### 2.2 Transcriptomic and Metabolomic Analyses Reveal that SkII Reprograms Serine Metabolism

**Figure 2:**
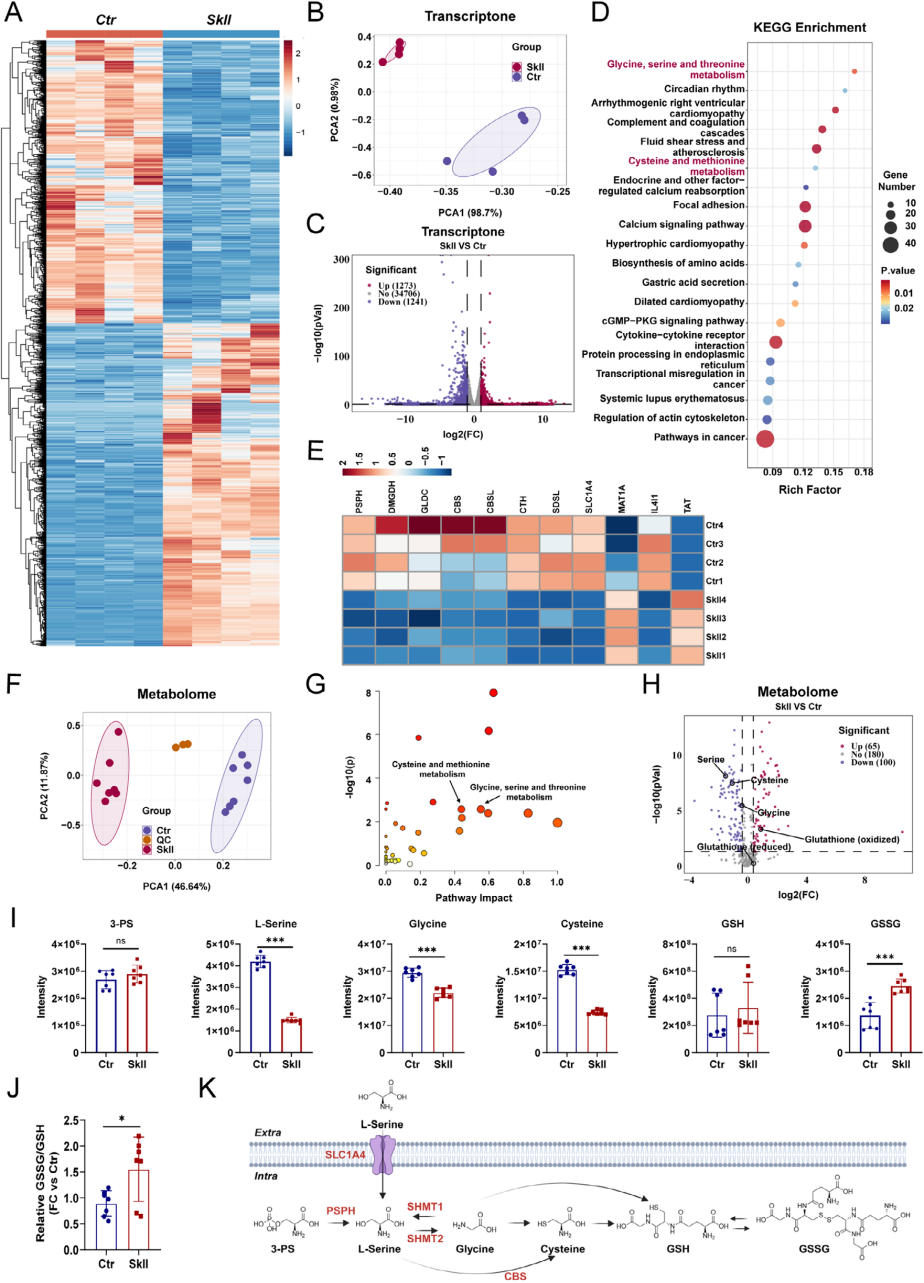
Transcriptomic and metabolomic analysis of HGC27 cells treated with SkII. A) Transcriptomic heatmap showing differentially expressed genes between the control and SkII (16 µM) treatment groups after 48 h (n = 4). B) PCA of transcriptomic data. C) Volcano plot showing differential gene expression in HGC27 SkII‐treated cells (16 µM) compared with control cells (upregulated, red; downregulated, blue; nonregulated, gray). The upregulated and downregulated genes were defined as those with *p* < 0.05 and FC ≥ 2. FC, fold change. D) KEGG enrichment analysis of differentially expressed genes from the transcriptomic data. E) Heatmap showing differentially expressed genes involved in the "glycine, serine, and threonine metabolism" and "cysteine and methionine metabolism" pathways in the transcriptome. F) PCA of the metabolomic profiles (Ctr group, n = 7; SkII group, n = 7; QC, n = 3). G) KEGG enrichment analysis of the differentially expressed metabolites. H) Volcano plot showiing differentially expressed metabolites in SkII‐treated cells (16 µM) compared with control cells (upregulated, red; downregulated, blue; nonregulated, gray). I) Abundances of 3‐PS, L‐serine, glycine, cysteine, GSH, and GSSG in the control group and the group treated with SkII (16 µM) for 48 h (n = 7). J) GSSG/GSH ratio between control and SkII (16 µM) treatment for 48 h (n = 7). K) Schematic representation of the core metabolites and related genes expressed from the transcriptome within the "glycine, serine, and threonine metabolism" and "cysteine and methionine metabolism" pathways. For all the statistical data, the data are presented as the means ± SDs, and statistical significance was determined by unpaired Student's t‐test. *Ns*, not significant; *p < 0.05, **p < 0.01, ***p < 0.001).

To further investigate the potential mechanisms by which SkII inhibits GC, we performed RNA sequencing (RNA‐seq) on HGC27 cells treated with DMSO (Ctr group) or 16 µM SkII (SkII group) for 48 h. A total of 37 220 RNA transcripts were detected, and 2514 transcripts were significantly altered in the SkII‐treated cells compared with the control group (Figure **2**A). Principal component analysis (PCA) revealed clear clustering of the four biological replicates within each group and distinct separation between the two groups (Figure 2B). Additionally, a volcano plot revealed the number of significantly upregulated (1273) and downregulated (1241) transcripts in the SkII group compared with the Ctr group (Figure 2C). KEGG enrichment analysis revealed the top 20 pathways that underwent substantial changes after SkII treatment (Figure 2D). Notably, we observed that SkII affected pathways related to glycine, serine, and threonine metabolism, as well as cysteine and methionine metabolism. A heatmap of the differentially expressed genes associated with these two metabolic pathways is shown in Figure 2E. Among these genes, the expression of the L‐serine transporter SLC1A4 was significantly reduced after SkII treatment, suggesting that SkII may interfere with serine metabolism at the level of amino acid transport.

Given that the transcriptomic analysis indicated that SkII affects intracellular amino acid metabolic processes, we performed metabolomic profiling of HGC27 cells treated with either SkII or DMSO using the Orbitrap Exploris 120 system. After quality control, a total of 345 metabolites were detected. PCA revealed significant differences in the metabolic profiles between the Ctr and SkII groups (Figure 2F). Additionally, pathway enrichment analysis of the metabolites using MetaboAnalyst revealed that SkII affects glycine, serine, and threonine metabolism, as well as cysteine and methionine metabolism, which is consistent with the transcriptomic findings (Figure 2G). Notably, SkII significantly reduced the abundances of L‐serine, glycine, and cysteine, while increasing the level of oxidized glutathione (GSSG), with no significant effect on the 3‐PS or reduced GSH levels (Figure 2H–J). The interactions among glycine, L‐serine, and threonine represent crucial metabolic crossroads. L‐serine is converted to glycine by serine hydroxymethyl transferase, with glycine entering the one‐carbon metabolism pathway. In one‐carbon metabolism, L‐serine and glycine drive the de novo synthesis of cysteine, which in turn is a key component in glutathione biosynthesis.^[^ ^26^ ^]^ Therefore, in this study, we define glycine, serine, and threonine metabolism along with cysteine and methionine metabolism as a single metabolic process, collectively summarized as serine metabolism. The metabolic pathways and interactions between these metabolites are illustrated in Figure 2K.

### 2.3 SkII Affects Serine Metabolic Flux in GC Cells by Inhibiting L‐serine Uptake

**Figure 3:**
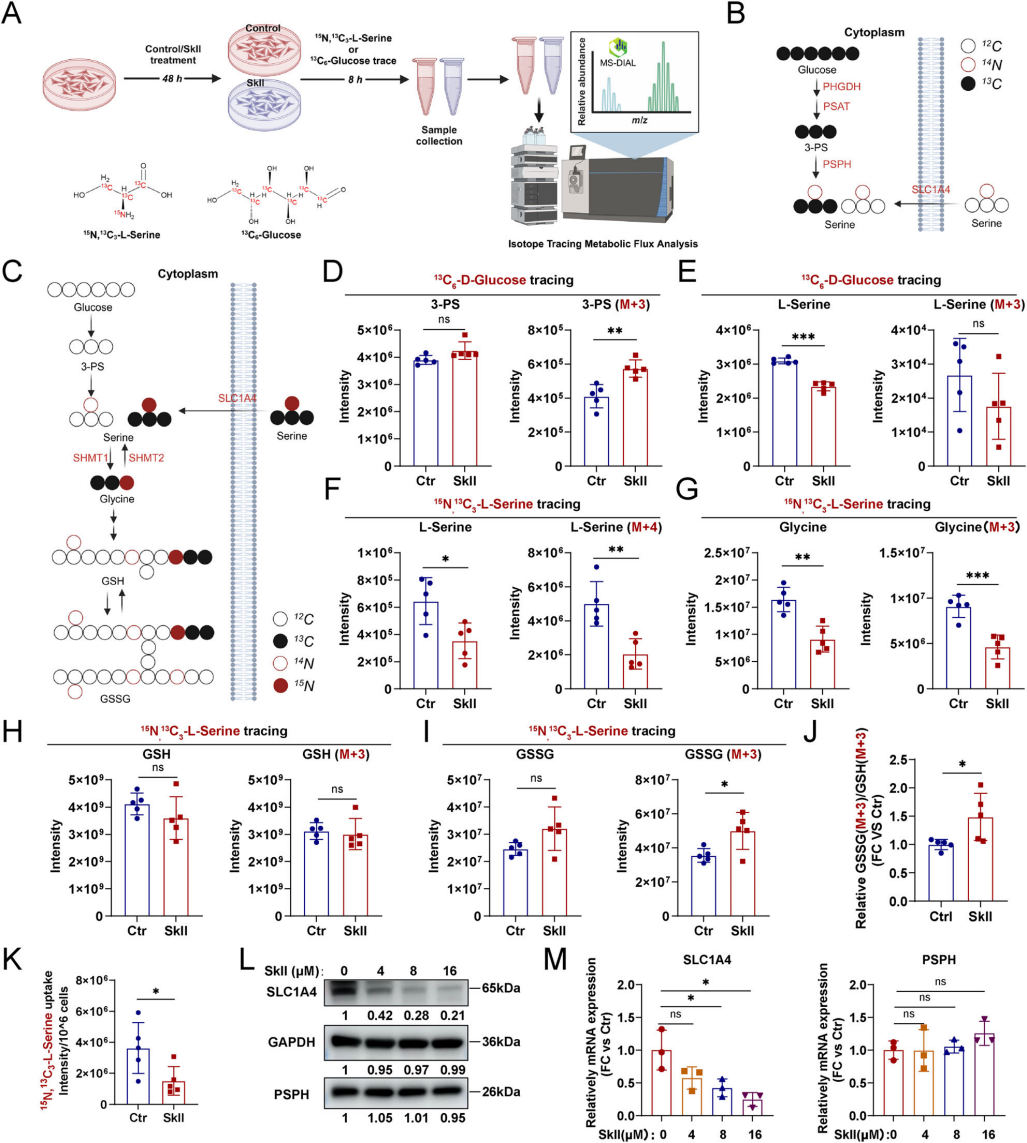
^13^C_6_‐D‐glucose and ^15^N,^13^C_3_‐L‐serine tracers reveal that SkII affects serine metabolic flux in GC cells. A) Schematic of the isotope tracing experiment. B) Schematic of ^13^C_6_‐D‐glucose administration to monitor L‐serine synthesis. C) Schematic of ^15^N,^13^C_3_‐L‐serine administration to monitor L‐serine uptake. D‐E) Stable quantification of the isotopolog abundances of 3‐PS and L‐serine in the HGC27 control and HGC27 SkII (16 µM) treatment group showing ^13^C_6_‐D‐glucose labeled carbon incorporation following 8 h of incubation in medium supplemented with ^13^C_6_‐D‐glucose (n = 5). F–I) Stable quantification of the isotopolog abundances of L‐serine, glycine, GSH and GSSG in the HGC27 control and HGC27 SkII (16 µM) treatment group showing ^15^N,^13^C_3_‐L‐serine labeled carbon and nitrogen incorporation following 8 h of incubation in medium supplemented with ^15^N,^13^C_3_‐L‐serine (n = 5). J) Ratio of GSSG to GSH incorporation in ^15^N,^13^C_3_‐L‐serine‐derived samples (n = 5). K) Stable quantification of the isotopolog abundances for ^15^N,^13^C_3_‐L‐serine uptake per 1 × 10^6^ HGC27 cells following 8 h of incubation in medium supplemented with ^15^N,^13^C_3_‐L‐serine (n = 5). L) Western blot analysis of SLC1A4, PSPH, and GAPDH (loading control) levels in HGC27 cells treated with SkII (0–16 µM) for 48 h. M) qRT‐PCR analysis of SLC1A4 and PSPH mRNA levels in HGC27 cells treated with SkII (0–16 µM) for 48 h, with GAPDH as an internal control (n = 3). For all the statistical data, the data are presented as the means ± SDs, and statistical significance was determined by one‐way ANOVA or unpaired Student's t‐test. *Ns*, not significant; *p < 0.05, **p < 0.01, ***p < 0.001.

Cellular L‐serine availability is regulated by two factors: the uptake of extracellular L‐serine and the de novo serine biosynthesis pathway, with low de novo serine synthesis capacity commonly observed in cancer.^[^ ^18^ ^]^ De novo serine biosynthesis is a branch of glycolysis, where L‐serine obtains carbon from glucose, while L‐serine uptake allows cancer cells to alternatively acquire L‐serine from the environment to replenish their intracellular level.^[^ ^27^ ^]^ To determine the primary mechanism by which SkII reduces intracellular L‐serine levels, we conducted isotope tracing experiments, as shown in **Figure** **3**A. These experiments utilized ^13^C_6_‐D‐glucose and ^15^N,^13^C_3_‐L‐serine (Figure 3B,C) as tracers to track the metabolic pathways contributing to serine metabolism. ^13^C_6_‐D‐glucose tracing revealed that SkII treatment significantly increased the abundance of ^13^C_3_‐3‐PS (Figure 3D), but had no significant effect on ^13^C_3_‐L‐serine levels (Figure 3E). Moreover, the m+3 isotope‐labeled fraction derived from ^13^C_6_‐D‐glucose significantly increased in 3‐PS, whereas SkII treatment had no effect on the m+3 isotope‐labeled fraction of L‐serine (Figure S2A,B, Supporting Information). These findings indicate that SkII does not affect intracellular L‐serine levels via the de novo biosynthesis pathway, increased 3‐PS intensity and isotope‐labeled fraction do not enter serine metabolism but rather affect other metabolic pathways. To test whether SkII acts by inhibiting L‐serine uptake, we used ^15^N,^13^C_3_‐L‐serine tracing. The results revealed that the intracellular ^15^N,^13^C_3_‐L‐serine and ^15^N,^13^C_2_‐glycine levels were significantly reduced following SkII treatment (Figure 3F,G), with downstream metabolites ^15^N,^13^C_2_‐GSH and ^15^N,^13^C_2_‐GSSG showing trends consistent with the metabolomics results (Figure 3H–J). After SkII treatment, the m+4 isotope‐labeled fraction derived from ^15^N,^13^C_3_‐L‐serine in intracellular L‐serine was significantly suppressed, whereas the m+3 isotope‐labeled fractions in GSH and GSSG significantly increased (Figure S2C–F, Supporting Information), suggesting that SkII may reduce the cellular uptake of exogenous labeled substrates by affecting transport proteins or inhibiting substrate uptake pathways. Furthermore, to confirm that SkII affects L‐serine uptake, we quantified the number of HGC27 cells and the corresponding abundance of extracellular ^15^N,^13^C_3_‐L‐serine. Compared with the control, SkII significantly reduced the amount of isotope‐labeled ^15^N,^13^C_3_‐L‐serine taken up by 1 × 10^6^ HGC27 cells (Figure 3K). Additionally, we examined the expression of the L‐serine uptake and de novo biosynthesis‐related transcripts, SLC1A4 and PSPH, from the transcriptomic data (Figure 2E). SkII treatment led to a dose‐dependent decrease in both SLC1A4 protein and mRNA levels, and markedly accelerated SLC1A4 degradation, while exerting no significant effect on PSPH (Figure 3L,M **and** Figure S2G, Supporting Information). Collectively, these results suggest that SkII reprograms serine metabolism by inhibiting L‐serine uptake rather than de novo serine biosynthesis.

### 2.4 SkII Directly Targets SLC1A4, Leading to Mitochondrial Damage

**Figure 4:**
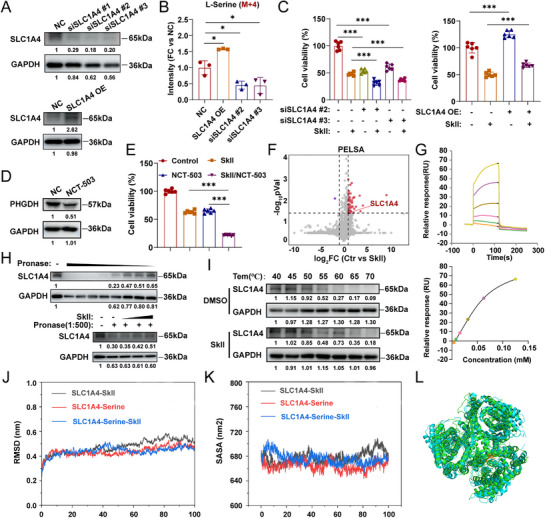
SLC1A4 was identified as the principal mediator of L‐serine uptake in GC and SkII directly targeted SLC1A4. A) Western blot analysis of SLC1A4 and GAPDH (loading control) in SLC1A4‐silenced (top) or SLC1A4‐overexpressing (bottom) HGC27 cells. B) L‐serine uptake levels in control, SLC1A4‐overexpressing HGC27 cells and SLC1A4‐silenced HGC27 cells following 8 h of incubation in medium supplemented with ^15^N,^13^C_3_‐L‐serine (n = 3). C) Viability of control, SLC1A4‐silenced and SLC1A4‐overexpressing HGC27 cells with or without SkII (16 µM) treatment for 48 h. D) Western blot analysis of PHGDH and GAPDH (loading control) in HGC27 cells with or without NCT‐503 (40 µM) treatment for 48 h. E) Viability of HGC27 cells treated with SkII, NCT‐503, or their combination for 48 h. F) Volcano plot visualizations of all proteins from a PELSA analysis of HGC27 lysates exposed to SkII. G) SPR analysis of the interaction between SkII and SLC1A4. H) DARTS assay and Western blot analysis of SLC1A4 and GAPDH (loading control) in HGC27 cells. Protease: protein ratios of 1:100, 1:200, 1:300, 1:400, 1:500, 1:1000, 1:2500, and 1:5000 were tested (top), with a protease: protein ratio of 1:500 selected for further experiments. Lysates were treated with various concentrations of SkII (4, 8, and 16 µM), and compared with DMSO‐treated controls (bottom). I) CETSA and Western blot analysis of SLC1A4 and GAPDH (loading control) following SkII or DMSO treatment. J) RMSD plot of SLC1A4‐SkII, SLC1A4‐Serine, SLC1A4‐SkII‐Serine. K) The SASA plot of SLC1A4‐SkII, SLC1A4‐Serine, SLC1A4‐SkII‐Serine. L) The structural conformation of the SLC1A4 protein in complex with L‐serine, in the presence (blue) or absence (green) of SkII. For all the statistical data, the data are presented as the means ± SDs, and statistical significance was determined by one‐way ANOVA. *Ns*, not significant; *p < 0.05, **p < 0.01, ***p < 0.001.

SLC proteins often exhibit broad substrate specificity, with many transporters capable of L‐serine uptake. In cases where a specific SLC is depleted, cells typically upregulate alternative SLCs to maintain L‐serine homeostasis. However, silencing certain SLC genes (e.g., SLC1A5 in breast cancer and SLC6A14 in colorectal cancer) can significantly impair L‐serine uptake, leading to metabolic disruption, as compensatory changes in other SLCs fail to sustain intracellular L‐serine levels. Although our transcriptomic and Western blot analyses confirmed that the L‐serine transporter SLC1A4 is regulated by SkII treatment, whether SLC1A4 is the dominant contributor to L‐serine uptake in GC cells and whether it is a direct target of SkII remain unclear. To functionally validate SLC1A4, we performed knockdown and overexpression experiments (Figure **4**A). Using ^15^N,^13^C_3_‐L‐serine tracing, we found that SLC1A4 knockdown reduced intracellular ^15^N,^13^C_3_‐L‐serine levels by more than 50% compared with those in control HGC27 cells, whereas SLC1A4 overexpression significantly increased L‐serine uptake, indicating that SLC1A4 plays a major role in L‐serine transport in GC cells (Figure 4B). Furthermore, SLC1A4 knockdown markedly decreased cell viability, and this effect was further exacerbated by SkII treatment. Conversely, SLC1A4 overexpression increased cell viability and attenuated the inhibitory effect of SkII (Figure 4C). To investigate the dependency on L‐serine uptake, we inhibited intracellular L‐serine synthesis using the PHGDH inhibitor NCT‐503 (Figure 4D), forcing cells to rely primarily on extracellular L‐serine uptake. Under these serine‐deprived conditions, the antitumor efficacy of SkII was significantly enhanced (Figure 4E).

To determine whether SkII directly modulates SLC1A4, we performed a peptide‐centric local stability assay (PELSA), which revealed that SkII alters the proteolytic sensitivity of SLC1A4, suggesting a direct interaction (Figure 4F). Surface plasmon resonance (SPR) assays using purified SLC1A4 protein confirmed that SkII binds SLC1A4 with a KD of 102 µM, indicating moderate affinity (Figure 4G). We further validated this interaction using drug affinity responsive target stability (DARTS). After screening protease concentrations (protease: protein = 1:100–1:5000), we selected a 1:500 ratio for subsequent experiments. Following incubation with SkII (4, 8, and 16 µM), SLC1A4 protein levels were greater than those in the DMSO control, indicating that SkII binding stabilizes SLC1A4 (Figure 4H). Additionally, a cellular thermal shift assay (CETSA) revealed that SLC1A4 degraded with increasing temperature, and that SkII protected SLC1A4 from thermal denaturation (Figure 4I). These results collectively support a direct interaction between SkII and SLC1A4, although additional targets may also contribute to its anticancer effects.

To elucidate how SkII binding affects the L‐serine transport function of SLC1A4, we performed molecular dynamics simulations (MD). The binding free energy between SLC1A4 and L‐serine was −19.88 kcal mol^−1^ (SASA: 655.74 nm^2^), whereas SLC1A4‐SkII binding exhibited a stronger affinity (−39.26 kcal mol^−1^, SASA: 679.99 nm^2^). In the presence of SkII, the binding free energy of SLC1A4‐L‐serine increased to −14.463 kcal mol^−1^, indicating a weakened interaction, whereas the SASA shifted to 677.94 nm^2^, reflecting altered surface properties (Figure 4J,K). Structural analysis revealed that SkII induces a more relaxed conformation in SLC1A4 (Figure 4L), providing mechanistic insight into how SkII allosterically inhibits SLC1A4‐mediated L‐serine transport.

**Figure 5:**
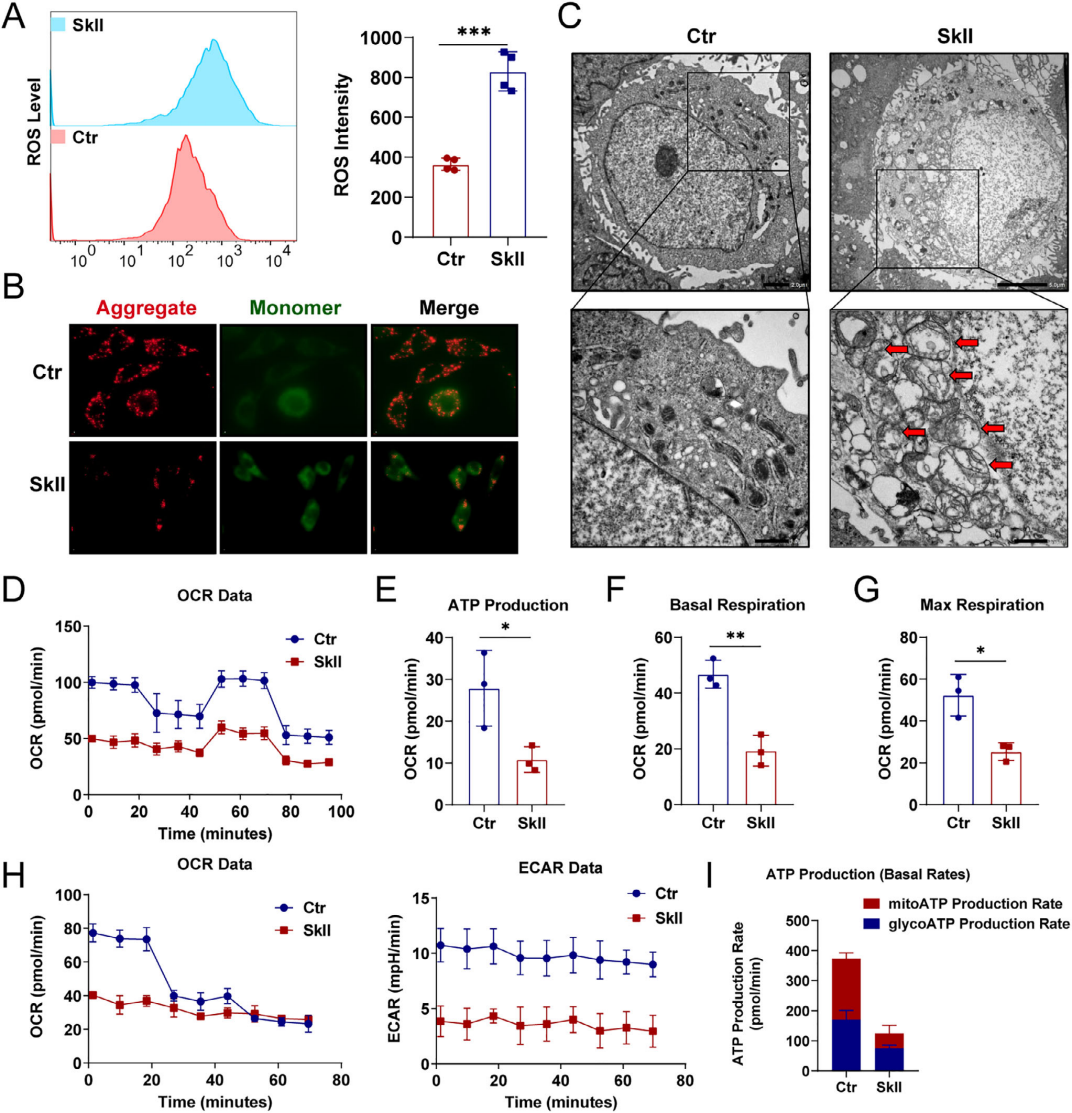
SkII increases mitochondrial ROS accumulation and inhibits mitochondrial energy metabolism to induce mitochondrial damage. A) Flow cytometry analysis of intracellular ROS levels in HGC27 cells with or without SkII (16 µM) treatment for 48 h. B) MMP was measured using a JC‐1 fluorescent probe in HGC27 cells with or without SkII (16 µM) treatment for 48 h. C) TEM analysis of the mitochondrial structure in HGC27 control or SkII (16 µM) treated cells for 48 h. D) Mitochondrial stress test in HGC27 cells treated with or without SkII (16 µM) for 48 h. E‐G) OCR analysis of ATP production, basal respiration, and maximal respiration in HGC27 control or SkII (16 µM) treated HGC27 cells after 48 h. H) Real‐time ATP rate assay in HGC27 control or SkII (16 µM) treated for 48 h. I) ATP production rate analysis in HGC27 control or SkII (16 µM) treated HGC27 cells for 48 h. For all the statistical data, the data are presented as the mean ± SDs, and statistical significance was determined by unpaired Student's t‐test. *Ns*, not significant; *p < 0.05, **p < 0.01, ***p < 0.001.

SkII directly binds to SLC1A4, inhibiting L‐serine uptake and ultimately leading to an increase in the GSSG/GSH ratio, which suggests that SkII may affect the intracellular redox balance. Therefore, we used flow cytometry to assess intracellular ROS levels, and the results revealed that SkII significantly increased ROS levels in HGC27 and AGS cells (Figures **5**A and S3A, Supporting Information). We employed the JC‐1 fluorescent probe to assess alterations in the mitochondrial membrane potential (MMP) in GC cells. Following SkII treatment, a marked reduction in red fluorescent aggregates was observed, indicating significant MMP disruption in treated cells (Figure 5B). The concomitant excessive accumulation of reactive oxygen species (ROS) and MMP dissipation collectively contribute to mitochondrial dysfunction, ultimately compromising cellular energy metabolism.^[^ ^28^ ^]^ Transmission electron microscopy (TEM) analysis revealed mitochondrial morphological damage in SkII‐treated GC cells, characterized by mitochondrial swelling and reduced crista density (Figures 5C and S3B, Supporting Information). Concurrently, the results of the mitochondrial stress test revealed that SkII‐treated GC cells presented lower basal oxygen consumption rates (OCR), with significant inhibition of basal respiration, ATP production, and maximal respiration (Figures 5D–G and S3C,D, Supporting Information). In addition, real‐time ATP rate assays revealed that the total ATP production rate in SkII‐treated GC cells was lower than that in control cells, with SkII significantly reducing both mitochondrial ATP and glycolytic ATP production rates (Figures 5H,I and S3E,F, Supporting Information). These results suggest that SkII primarily inhibits the cellular energy supply by inducing oxidative stress and mitochondrial damage.

### 2.5 SkII Exhibits Antitumor Efficacy and Inhibits Intratumoral Serine Metabolism In Vivo

**Figure 6:**
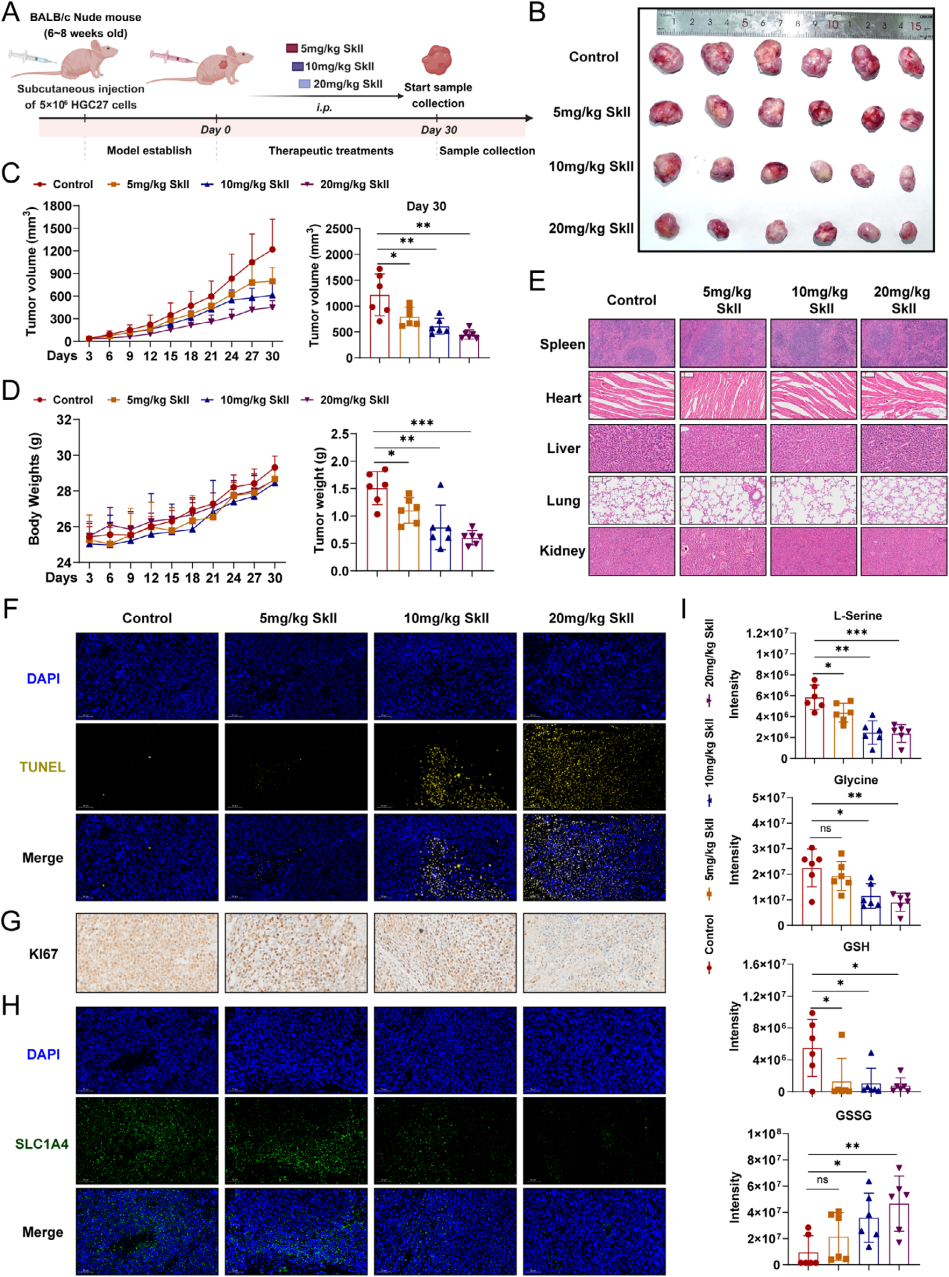
SkII reduces serine metabolism in tumors by inhibiting SLC1A4 expression. A) Experimental scheme for establishing a subcutaneous xenograft tumor model in mice. B) Representative images of tumors isolated from mice (n = 6). C) Growth curves of tumor volume in mice during SkII treatment (left), and tumor volumes on day 30 (right). D) Changes in body weight during SkII treatment (left), and isolated tumor weight (right). E) H&E staining of heart, liver, spleen, lung, and kidney tissues from mice after SkII treatment. F) Representative IHC images of TUNEL staining in tumors at different concentrations of SkII. G) Representative IHC images of KI67 expression in tumors at different concentrations of SkII. H) Representative IHC images of SLC1A4 expression in tumors at different concentrations of SkII. I) Abundances of L‐serine, glycine, GSH, and GSSG in tumors at different concentrations of SkII. (n = 6) For all the statistical data, the data are presented as the mean ± SDs, and statistical significance was determined by one‐way ANOVA. *Ns*, not significant; *p < 0.05, **p < 0.01, ***p < 0.001.

To evaluate whether SkII has therapeutic effects on GC tumor growth in vivo, HGC27 cells were subcutaneously injected into BALB/c nude mice to establish a subcutaneous tumor model. The mice were randomly divided into a control group and SkII groups (5, 10, and 20 mg kg^−1^), with intraperitoneal injections of vehicle or SkII administered every other day for 30 days (Figure **6**A). Compared with the control treatment, SkII treatment significantly inhibited subcutaneous tumor growth, as the tumor volume and weight were markedly reduced (Figure 6B–D). Additionally, the mice tolerated the administered doses of SkII well, and H&E staining revealed no damage to the heart, liver, spleen, lung, or kidney tissues of the tumor‐bearing mice following SkII treatment (Figure 6E). To investigate the mechanisms underlying SkII‐mediated tumor growth inhibition, we performed immunohistochemical (IHC) staining to assess the effects of SkII on apoptosis and proliferation. TUNEL and KI67 staining indicated that SkII suppressed tumor growth by inducing apoptosis and inhibiting cell proliferation (Figure 6F,G). Furthermore, SLC1A4 was highly expressed in tumor tissues, and SkII reduced SLC1A4 protein levels in a dose‐dependent manner (Figure 6H). Consistent with the metabolomics results observed in cells, SkII treatment significantly reduced the levels of L‐serine, glycine, and GSH in tumor tissues while increasing the GSSG level (Figure 6I). These findings suggest that SkII significantly reduces the SLC1A4 level, induces apoptosis, inhibits proliferation, and suppresses serine metabolism.

### 2.6 Dietary Restriction of Serine and Glycine Enhances the Tumor‐Suppressive Effects of SkII

**Figure 7:**
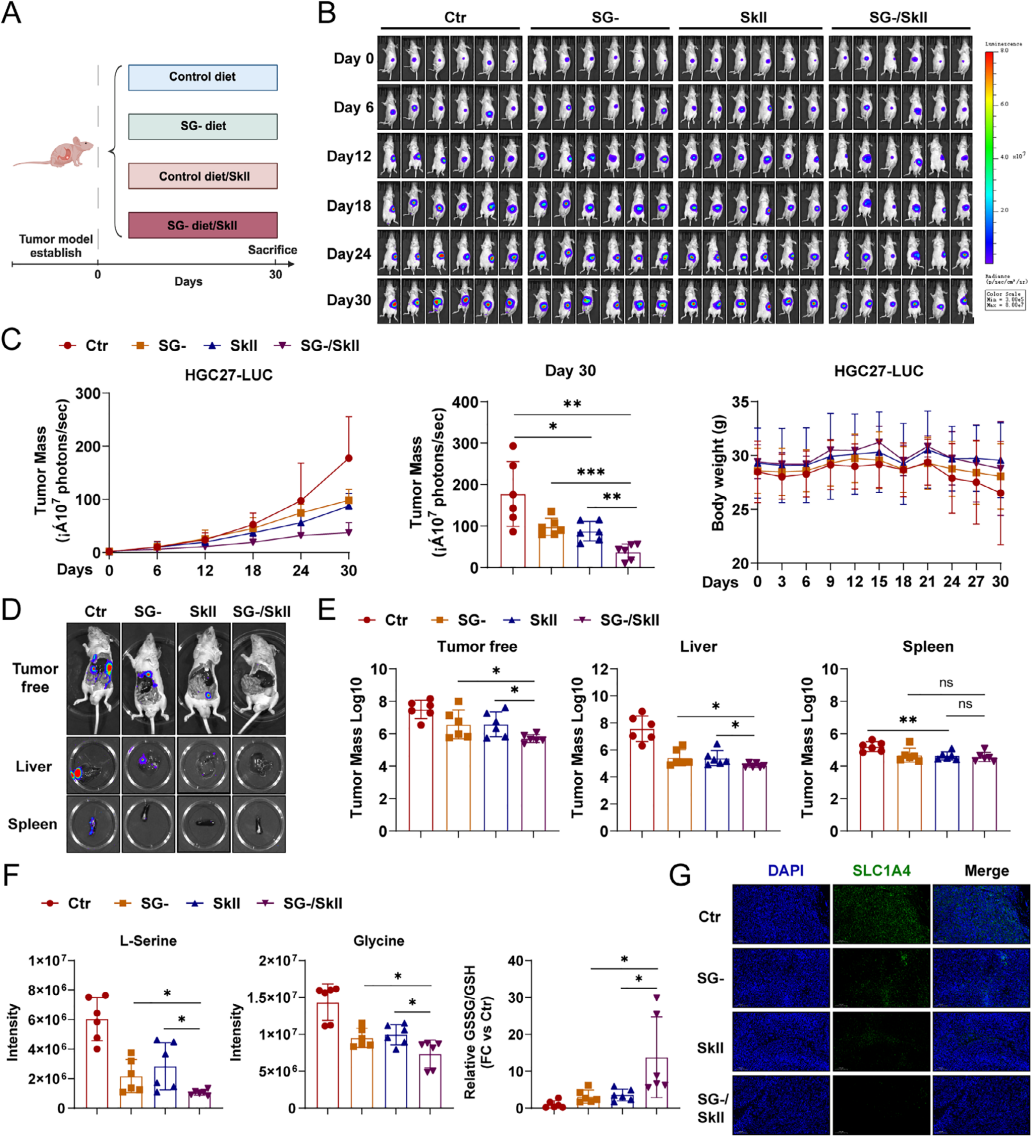
Serine and glycine dietary restriction enhances the tumor‐suppressive effects of SkII. A) Experimental scheme for establishing an orthotopic tumor model and feeding strategies in mice. B) Representative fluorescence images of orthotopic tumors in mice at different time points in each group (n = 6). C) Quantification of the tumor mass fluorescence intensity in mice at different time points, and changes in body weight. D) Representative images of tumor free, spleen, and liver metastases in mice. E) Quantification of tumor mass fluorescence intensity in the tumor free, spleen, and liver metastases in mice. F) Abundances of L‐serine, and glycine, and the ratio of GSSG to GSH in each group. G) Representative IHC images of SLC1A4 expression in tumors from each group. For all the statistical data, the data are presented as the mean ± SDs, and statistical significance was determined by one‐way ANOVA. Ns, not significant; *p < 0.05, **p < 0.01, ***p < 0.001.

Our in vitro experiments demonstrated that SkII inhibits L‐serine uptake by directly targeting the L‐serine transporter protein SLC1A4, thereby affecting serine metabolism in GC cells. Therefore, we fed orthotopic tumor‐bearing mice either a normal diet or a serine and glycine deficient (SG‐) diet (in which serine and glycine are interconverted via serine hydroxymethyl transferase) to assess the effects of SkII treatment (Figure **7**A). The live imaging results indicated that tumor growth was inhibited by the SG‐ diet, and that SkII treatment enhanced this effect. Toxicity was evaluated by monitoring body weight changes. Compared with the control group, neither the SG‐ diet alone nor the combination of SkII and the SG‐ diet caused significant changes in body weight (Figure 7B,C). Metastasis is a critical issue in cancer, particularly in GC, where metastases commonly occur in the liver and peritoneum.^[^ ^29^ ^]^ We investigated the effects of the SG‐ diet or the combination of SkII with the SG‐ diet on metastasis to the liver, peritoneum, and spleen. The results revealed that both the SG‐ diet and the SkII treatment effectively suppressed metastatic tumor nodules in the spleen and liver, with the combination of SkII and the SG‐ diet further enhancing this effect (Figure 7D,E and Figure S4A–C, Supporting Information). The SG‐ diet significantly suppressed intratumorally L‐serine and glycine levels while exacerbating oxidative stress. Notably, coadministration of SkII with the SG‐ diet potentiated these antitumor effects through synergistic modulation of both metabolic and oxidative pathways (Figure 7F). In addition, SLC1A4 expression was also correspondingly reduced (Figure 7G).

### 2.7 SLC1A4 is Upregulated in GC Tissues and Inhibited by SkII

**Figure 8:**
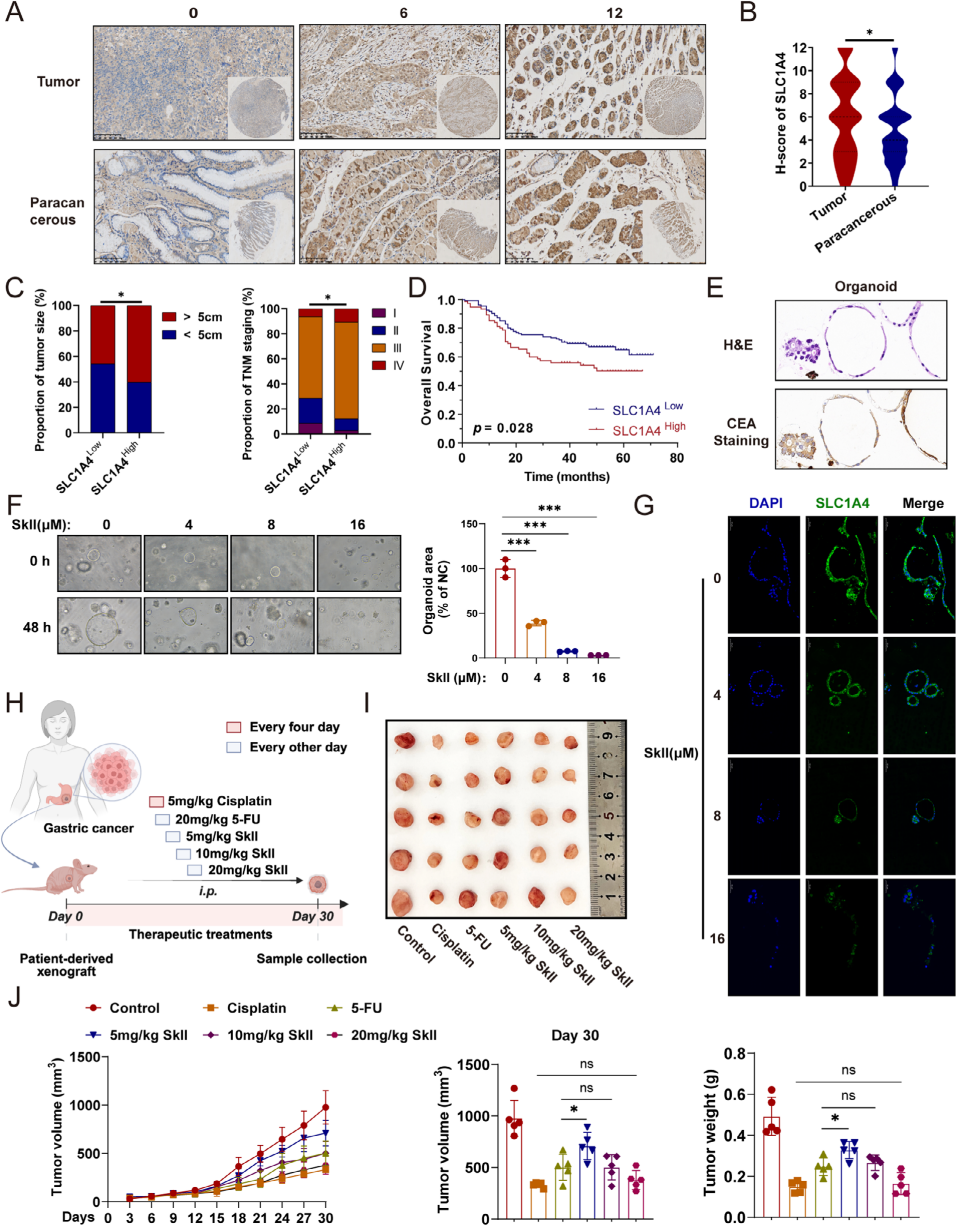
SLC1A4 overexpression drives gastric tumorigenesis and serves as a therapeutic target of SkII. A) Representative IHC images of various SLC1A4 expression in GC tumors and adjacent paracancerous tissues. B) SLC1A4 expression levels were significantly higher in GC tumor tissues than in paracancerous tissues. C) A percentage‐stacked bar chart illustrating the distribution of tumor sizes (left), and TNM stages (right) between the SLC1A4 high and SLC1A4 low‐expression groups. D) Kaplan‐Meier survival analysis showing the relationship between SLC1A4 expression and prognosis in GC patients. E) Representative images of H&E staining and IHC of the GC protein marker CEA in GC organoids. F) Representative images of GC organoids (left) and organoids growth (right) after treating with SkII (0–16 µM) for 48 h. G) Representative IHC images of SLC1A4 expression in organoids treated with SkII (0–16 µM) for 48. H) Experimental scheme for establishing a PDX in mice. I) Representative images of tumors isolated from mice treated with 5‐FU, cisplatin or different concentrations of SkII (n = 5). J) Tumor volumes growth curves and tumor weights of mice treated with 5‐FU, cisplatin or different concentrations of SkII. For all the statistical data, the data are presented as the mean ± SDs, and statistical significance was determined by one‐way ANOVA. *Ns*, not significant; *p < 0.05, **p < 0.01, ***p < 0.001.

To verify the overexpression of the SLC1A4 protein in GC, we examined its expression in GC tissues using IHC staining of tissue microarrays and analyzed its correlation with clinicopathological characteristics. In this study, an H‐score of 6 was used as the cutoff value, with H‐scores>6 classified as the SLC1A4‐high expression group and H‐scores≤6 as the SLC1A4‐low expression group. Figure **8**A presents representative IHC staining images for SLC1A4. SLC1A4 expression was significantly higher in GC tissue samples than in the paracancerous tissue samples (Figure 8B). Moreover, the expression level of SLC1A4 was strongly associated with tumor size and TNM stage, suggesting that SLC1A4 may have contributed to the progression of GC (*P*<0.05, Figure 8C and Table S1, Supporting Information). High SLC1A4 expression levels were also associated with shorter overall survival (OS) in patients with GC (Figure 8D). GC organoids retain the histological characteristics observed in their corresponding primary tumors. Therefore, we treated GC organoids with SkII to model the potential effects of SKII on primary GC. To validate whether GC organoids retain the phenotypic characteristics of their parental tumors, we performed IHC analysis of the GC biomarker carcinoembryonic antigen (CEA). The results demonstrated strong CEA expression in the GC‐derived organoids (Figure 8E), confirming their faithful maintenance of the original tumor characteristics. Consistent with the in vitro observations, SkII treatment effectively inhibited the growth of GC organoids (Figure 8F). Immunofluorescence analysis confirmed that SkII administration significantly suppressed SLC1A4 expression in GC organoids (Figure 8G). These findings suggest that SkII inhibits the growth of GC organoids by downregulating SLC1A4 expression. To compare the therapeutic efficacy of SkII with that of first‐line clinical agents (cisplatin and 5‐FU), we employed PDX models (Figure 8H). Our results demonstrated that, compared with 5‐FU (20 mg kg^−1^), SkII (10 mg kg^−1^) achieved comparable tumor growth inhibition, whereas the antitumor efficacy of SkII (20 mg kg^−1^) was similar to that of cisplatin (5 mg kg^−1^) (Figure 8I,J). Collectively, these findings substantiate the clinical translational potential of SkII as a therapeutic candidate.

## 3 Discussion

GC is a highly malignant and aggressive gastrointestinal tumor that represents a significant contributor to the global cancer burden.^[^ ^30^ ^]^ In the search for effective anti‐GC candidate compounds, traditional Chinese herbs and their natural compounds have shown great potential. Flavonoids, the most common natural compounds in traditional herbs, consist of three central carbon atoms that form a C6‐C3‐C6 unit. Recent studies have increasingly demonstrated the beneficial effects of flavonoids in the inhibition of GC.^[^ ^31^ ^]^ In this study, we demonstrate for the first time that SkII, a flavonoid compound derived from *Scutellaria*, exhibits selective cytotoxicity against GC cells and inhibits their proliferation and metastasis both in vitro and in vivo. Further investigation revealed that SkII targets and inhibits the L‐serine transporter protein SLC1A4, leading to reduced intracellular levels of L‐serine, glycine, and their downstream metabolites. This, in turn, results in excessive ROS accumulation, mitochondrial damage, and disruption of cellular energy metabolism. Furthermore, the organoids and PDX models maintain the heterogeneity and complexity of the original tumors, thereby enabling accurate simulation of patient‐specific drug responses.^[^ ^32^ ^]^ SkII consistently demonstrated robust anti‐GC efficacy across both the organoids and PDX models. Our findings support the potential of serine metabolism inhibitors, such as SkII, as therapeutic options for GC.

Metabolic reprogramming is a hallmark of cancer cells, and the metabolic vulnerabilities of cancer cells represent promising targets to increase therapeutic efficacy and cure rates. Notably, cancer cells are highly dependent on serine metabolism, as serine metabolism supports cell proliferation and survival processes.^[^ ^33^ ^]^ Several studies have also reported therapeutic advantages of targeting serine metabolism to impair tumor growth and enhance the efficacy of anticancer drugs.^[^ ^6^ ^]^ In this study, using metabolomics and transcriptomics, we found that SkII treatment inhibits serine metabolism in GC cells. L‐serine is involved in several key metabolic processes, including protein biosynthesis, GSH synthesis, and one‐carbon metabolism.^[^ ^34^ ^]^ L‐serine‐derived glycine and cysteine are components of GSH, and under L‐serine‐limited conditions, cancer cells prioritize GSH synthesis to maintain antioxidant defenses. Disruption of serine metabolism within mitochondria also affects the GSH/GSSG ratio.^[^ ^35^ ^]^ To determine whether the inhibition of serine metabolism by SkII affects redox balance in GC cells, we measured the intracellular levels of GSH, GSSG, and ROS. Interestingly, SkII treatment did not alter GSH levels but led to the accumulation of GSSG and ROS, which in turn increased mitochondrial stress, promoted mitochondrial damage, and disrupted mitochondrial energy metabolism.

Serine depletion significantly inhibits cancer cell growth, and intracellular L‐serine can be acquired through various transporters or synthesized de novo by cells. Although the activation of de novo serine biosynthesis in cancer was described nearly 30 years ago, recent identification of PHGDH amplification or overexpression in breast cancer and melanoma has reignited interest in the role of this pathway in tumorigenesis.^[^ ^36^ ^]^ Cancer cells with high PHGDH expression exhibit increased levels of de novo serine biosynthesis and, unsurprisingly, have a reduced dependency on exogenous L‐serine for growth. Furthermore, dietary restriction of serine and glycine can suppress tumor growth and improve survival in various genetically engineered mouse models of cancer. Metabolite analysis of a panel of 60 cancer cell lines revealed a strong preference for consuming extracellular L‐serine, with L‐serine being the second most consumed amino acid after glutamine.^[^ ^37^ ^]^ In this study, SkII treatment was shown to decrease L‐serine levels in GC cells. To further elucidate whether this effect was due to an impact on the de novo serine biosynthesis pathway or L‐serine uptake, we employed isotope tracers to investigate the underlying mechanism. The results confirmed that the inhibition of intracellular L‐serine levels by SkII was entirely due to its effect on L‐serine uptake, as neither the levels of PSPH, a key protein in de novo serine synthesis, nor L‐serine derived from ^13^C_6_‐glucose were affected by SkII. Furthermore, to explore the role of L‐serine in the physiological environment in vivo, we fed HGC27 orthotopic tumor‐bearing mice either a control diet or a SG‐ diet. We found that tumor size in SG‐ diet‐fed mice was inversely correlated with the duration of SG‐ diet consumption, and this dietary intervention enhanced the antitumor effect of SkII treatment.

Amino acid transport in mammalian cells is mediated primarily by secondary active transporters of the SLC family. Tumor growth can significantly influence the expression and function of SLC transporters through disruptions in physiological amino acid homeostasis. In several tumor cells, where de novo serine biosynthesis capacity is limited, the maintenance of intracellular L‐serine homeostasis predominantly relies on extracellular L‐serine uptake mediated by SLC transporters.^[^ ^10^ ^]^ Although tumor cells typically modulate multiple SLC transporters for L‐serine acquisition, one primary SLC transporter usually serves as the major contributor to the L‐serine supply. Notably, the deficiency of this dominant transporter cannot be compensated for by other SLC family members to restore intracellular L‐serine homeostasis. In this study, we observed that SLC1A4 expression was elevated in GC tissues compared with adjacent non‐cancerous tissues, and that SLC1A4 serves as the predominant contributor to L‐serine uptake in GC cells. SkII treatment significantly reduced SLC1A4 levels in both mouse tumor tissues and human GC organoids. Based on results of MD, DARTS, CETSA, and SPR assays, we propose that SkII inhibits L‐serine uptake by binding to SLC1A4 and thereby impairing its function.

Overall, the findings of this study demonstrate that targeting SLC1A4 is a key mechanism by which SkII disrupts serine metabolism, thereby reducing intracellular L‐serine levels in GC cells by inhibiting uptake rather than de novo synthesis. This metabolic disruption leads to mitochondrial damage, impaired energy production, and increased apoptosis in GC cells. These results highlight the potential of SkII as a promising therapeutic candidate for patients with GC. Notably, although our findings support SkII exerts its anticancer effects by targeting SLC1A4 to inhibit L‐serine uptake, we acknowledge that the molecular mechanisms underlying SkII‐induced SLC1A4 degradation remain incompletely understood. Future studies will therefore be required to clarify the downstream regulatory pathways mediating this process. Current data also indicate that SkII may act on other targets and participate in broader metabolic and signaling pathway regulation. Therefore, while inhibition of SLC1A4 represents a key pathway for antitumor activity of SkII in gastric cancer, it is not the sole mechanism. Future studies are warranted to further explore additional potential targets of SkII and its integrated network of actions to comprehensively elucidate its anticancer effects.

## 4 Experimental Section

### Reagents

Skullcapflavone II (Cat# TN1040) was purchased from TargetMol (Boston, MA, USA). Isotopically labeled ^13^C_6_‐D‐glucose (Cat# CLM‐1396) was obtained from Cambridge Isotope Laboratories (Woburn, USA), and ^15^N,^13^C_3_‐L‐serine (Cat# 1ST1416C3N) was purchased from Alta Scientific Co., Ltd. (Tianjin, China). M‐PER Reagent (Cat# 78505), LC‐MS‐grade acetonitrile and methanol, as well as HPLC‐grade formic acid were obtained from Thermo Fisher Scientific (Waltham, MA, USA). The ACQUITY UPLC HSS T3 Column (Cat# 186003539) was purchased from Waters (Milford, MA, USA). Ultrapure water was supplied by Wahaha Co., Ltd. (Hangzhou, China). The SLC1A4 human recombinant protein (Cat# TP304487) was purchased from OriGene Technologies, Inc. (Delaware, USA). TRIzol reagent (Cat# 15596026CN) and MitoSOX Red mitochondrial superoxide indicator (Cat# M36008) were purchased from Invitrogen (Carlsbad, CA, USA). Pronase (Cat# PRON‐RO) was obtained from Merck Millipore (Billerica, MA, USA), and D‐Luciferin, Sodium Salt D (Cat# 40901ES08‐EN) was purchased from Yeasen Biotechnology Co., Ltd. (Shanghai, China). The Seahorse XFe24 FluxPak mini (Cat# 102342‐100), Seahorse XF Real‐Time ATP Rate Assay Kit (Cat# 103592‐100), and Seahorse XF Cell Mito Stress Test Kit (Cat# 103015‐100) were purchased from Agilent Technologies (Santa Clara, CA, USA). The RNA Quick Purification Kit (Cat# RN001) was purchased from Shanghai Yishan Biotechnology Co., Ltd. (Shanghai, China). The Bradford Protein Assay Kit (Cat# P0006C), Annexin V‐FITC Apoptosis Detection Kit (Cat# C1062), and EdU Cell Proliferation Kit (Cat# C0078) were purchased from Beyotime (Shanghai, China). The 6.5 mm Transwell with 8.0 µm pore polycarbonate membrane inserts (Cat# 3422) were purchased from Corning (New York, USA), and Culture‐Inserts 4 Well for self‐insertion (Cat# 80469) were obtained from Ibidi (Martinsried, Germany). Cell culture dishes/plates were obtained from NEST Biotechnology Co., Ltd (Wuxi, China). Primary antibodies against N‐cadherin (Cat# 22018‐1‐AP), E‐cadherin (Cat# 20874‐1‐AP), MMP2 (Cat# 10373‐2‐AP), Vimentin (Cat# 10366‐1‐AP), GAPDH (Cat# 60004‐1‐Ig), Bax (Cat# 50599‐2‐Ig), Bcl‐2 (Cat# 12789‐1‐AP), SLC1A4 (Cat# 13067‐2‐AP), and PSPH (Cat# 14513‐1‐AP) were purchased from Proteintech (Wuhan, China). Primary antibodies against PARP (Cat# 9532) and cleaved PARP (Cat# 5625) were obtained from Cell Signaling Technology (Danvers, MA, USA). RT Master Mix for qPCR (Cat# HY‐K0511) and SYBR Green qPCR Master Mix (Cat# HY‐K0523) were purchased from MedChem Express (Monmouth Junction, NJ, USA). SG‐ diet was obtained from Xietong Medical Biotechnology Co., Ltd. (Nanjing, China), and the composition of the diet is listed in Table S2, Supporting Information.

### Patients and Sample Collection

From August 2014 to August 2017, 210 GC patients undergoing treatment at Zhejiang Cancer Hospital were recruited for this study. H&E stained sections were prepared from surgically excised tumor tissues from each patient. Paracancerous tissues were defined as samples obtained 2 cm from the tumor margin, and corresponding paracancerous tissues were collected adjacent to the tumor specimens. Histological evaluation of all available slides from the enrolled patients was independently performed by two pathologists following standardized protocols. In total, 210 tumor tissue samples and 150 paracancerous tissue samples were obtained for analysis.

To assess SLC1A4 expression, the H‐score method was used, which was calculated as H‐score = IS × AP, where IS represents the intensity of staining and AP denotes the proportion of positively stained cells. IS was scored as follows: 0, no staining; 1, weak staining; 2, moderate staining; and 3, strong staining. AP was categorized as: 0 for 0%; 1 for 1–25%; 2 for 26–50%; 3 for 51–75%; and 4 for 76–100%. Patients were stratified into high and low SLC1A4 expression groups based on the median H‐score.

### Cell Culture

The human cell lines GES‐1, HGC27, and AGS were purchased from the American Type Culture Collection (ATCC, USA) and cultured following the manufacturer's protocols.

### Animal Experiments and Treatment

Male BALB/c nude mice (6–8 weeks old, weighing 22 ± 2 g) were purchased from GemPharmatech Co., Ltd. (Shanghai, China) and acclimatized for 1 week prior to use. All mice were maintained in SPF‐grade animal facilities with a 12 h light/dark cycle. All mice care and experimental procedures were conducted in accordance with the guidelines approved by the Animal Care and Use Committee of the Hangzhou Institute of Medicine, Chinese Academy of Sciences (Approval No. AP2024‐12‐0394).

For the subcutaneous xenograft tumor model, HGC27 cells (5 × 10^6^ cells/100 µL) were injected into the left forelimb of the mice. When tumors became palpable, the mice were randomly divided into four groups (6 mice per group) and administered different doses of SkII or vehicle intraperitoneally every other day. The tumor volume was measured with calipers and calculated using the following formula: volume = length × width^2^/2. After the final treatment, the mice were euthanized, and the tumor tissue was excised and weighed.

For the orthotopic xenograft tumor model, HGC27‐LUC cells (5 × 10^6^ cells/100 µL) were first injected into the left forelimb of the mice. When the tumor volume reached 500 mm^3^, the mice were euthanized, and the tumors were excised. The tumor was then cut into 4–5 mm^3^ fragments and surgically implanted into the stomachs of the mice. The success of tumor formation was confirmed using the Caliper Life Science IVIS Lumina II imaging system. Successful model mice were randomly assigned to four groups (6 mice per group) and fed either a normal diet or a SG‐ diet. They received intraperitoneal injections of SkII (10 mg kg^−1^) or vehicle every other day. Bioluminescence was monitored every 6 days, and after the final treatment, the mice were euthanized. Tumor free, liver and spleen tissues were excised for imaging to observe tumor metastasis in the abdominal cavity. All imaging results were processed using Living Image 4.4 software.

For the patient‐derived xenograft tumor model, tumor tissue obtained from patients with GC was subcutaneously transplanted into nude mice and serially passaged for more than 3 generations. The tumor tissues were then dissected into 4–5 mm^3^ fragments and surgically implanted into the left forelimb of recipient mice. Mice were randomly divided into 6 groups (n = 5 per group) and administered cisplatin (5 mg kg^−1^) every 4 day, administered 5‐FU (20 mg kg^−1^) and different doses of SkII intraperitoneally every other day. Cisplatin (5 mg kg^−1^) and 5‐FU (20 mg kg^−1^) were administered based on clinically relevant doses. After the final treatment, the mice were euthanized, and the tumor tissue was excised and weighed.

### Transmission Electron Microscopy

Following treatment with various concentrations of SkII, cells were collected by trypsinization and fixed with 2.5% glutaraldehyde. The samples were rinsed 3–4 times with 0.1 M phosphate buffer for 15 min each, followed by fixation with 1% osmium tetroxide. After dehydration through a graded ethanol series and resin infiltration, the samples were embedded and sectioned into ultrathin slices (50–70 nm). The sections were stained with 2% uranyl acetate for 10–30 min and lead citrate for 5–15 min. Imaging was performed using a transmission electron microscopy (JEM‐2100plus).

### Flow Cytometry Assays

HGC27 and AGS cells were cultured at a density of 2 × 10⁵ cells per well in 6‐well plates. After overnight incubation, the cells were treated with various concentrations of SkII (0, 4, 8, and 16 µM) for 48 h. The cells were then collected by trypsinization and stained for apoptosis using an Annexin V‐FITC Apoptosis Detection Kit, following the manufacturer's instructions. The intracellular ROS levels were determined by staining according to the MitoSOX Red protocol. Flow cytometry data were acquired using the Attune NxT flow cytometer and analyzed with FlowJo software.

### Molecular Dynamics Simulations

Molecular dynamics simulations were performed using the Amber24 software. The ff19SB force field and OPC water model were employed to solvate the complex in a cubic water box. A time step of 2 fs was used, and the system was maintained at 300 K and 1 bar using the V‐rescale thermostat and Parrinello‐Rahman barostat. After energy minimization, the system underwent a 300 ps equilibration protocol (200 ps NVE followed by 100 ps NPT), followed by a 100 ns production simulation. RMSD and SASA were performed using built‐in Amber modules.

### EdU Assay

HGC27 and AGS cells were cultured at a density of 5 × 10⁴ cells per well in 12‐well plates. After overnight incubation, the cells were treated with various concentrations of SkII (0, 4, 8, and 16 µM) for 48 h. Cell proliferation was assessed by staining with an EdU Cell Proliferation Kit according to the manufacturer's instructions. Fluorescence images were captured using an Olympus CKX53 inverted fluorescence microscope.

### Colony Formation Assay

HGC27 and AGS cells were seeded at a density of 1000 cells per well in 6‐well plates. After overnight incubation, the cells were treated with various concentrations of SkII (0, 4, 8, and 16 µM) for 48 h. The SkII‐containing medium was then removed, and the cells were cultured in complete medium for 10 days in a humidified incubator at 37 °C with 5% CO_2_, with fresh complete medium replaced every 1–2 days to allow colony formation. Colonies were fixed with 4% paraformaldehyde and stained with 0.1% crystal violet.

### Wound Healing Assay

HGC27 and AGS cells were seeded at a density of 1 × 10⁵ cells per chamber in 4‐well culture‐inserts. After overnight incubation, the inserts were removed, and 0‐h images were captured with an Olympus CKX53 inverted fluorescence microscope. The cells were then treated with various concentrations of SkII (0, 4, 8, and 16 µM) in RPMI 1640 medium containing 2% FBS for 48 h, followed by the capture of 48‐h images.

### qRT‐PCR Analysis

Total RNA was extracted using the RNA Quick Purification Kit. Reverse transcription was performed using the RT Master Mix for qPCR. qPCR was carried out using the SYBR Green qPCR Master Mix on a CFX96 Real‐Time System (Bio‐Rad Laboratories). Relative gene expression levels were calculated using the 2^‐ΔΔCt^ method and normalized to the expression of the internal control, GAPDH. All primers used are listed in Table S3, Supporting Information.

### Transwell Migration and Invasion Assays

After digestion, HGC27 and AGS cells were resuspended in serum‐free medium. A total of 200 µL of cell suspension was added to the upper chamber (coated or uncoated with Matrigel), while 700 µL of RPMI 1640 medium containing 10% FBS was added to the lower chamber. The cells were treated with various concentrations of SkII (0, 4, 8, and 16 µM) for 48 h. After treatment, cells in the upper chamber were removed with a cotton swab, and the cells in the lower chamber were fixed with 4% paraformaldehyde and stained with crystal violet. Images of the cells in the lower chamber were captured.

### Western Blot

Total protein was extracted from cells using M‐PER Reagent, and the protein concentration was quantified using a Bradford Protein Assay Kit for normalization. Protein samples were separated via SDS‐PAGE and transferred to a PVDF membrane. The membrane was washed 3 times with TBST, following by overnight incubation at 4 °C with the primary antibody. Following incubation, the membrane was washed again and then incubated with the secondary antibody. Protein bands were visualized using an ECL detection kit (Cat# P10100, New Cell & Molecular Biotech).

### Seahorse Assays

Metabolic data on energy production in cells were analyzed using an Agilent Seahorse XFe 24 analyzer. HGC27 and AGS cells were seeded at a density of 6000 cells per well in the Seahorse XFe24 FluxPak. After culturing, the cells were treated with various concentrations of SkII (0 and 16 µM) for 48 h. Prior to analysis, the original medium was removed and replaced with Seahorse XF RPMI 1640 containing 2 mM L^−1^ glutamine, 10 mM/ glucose, and 2 mM L^−1^ sodium pyruvate. The cells were incubated in a non‐CO_2_ incubator at 37 °C for 1 h to establish equilibrium before loading. ATP production rates were measured over time using the Agilent Seahorse Real‐Time ATP Rate Assay Kit, and mitochondrial stress was assessed using the Agilent Seahorse XF Cell Mito Stress Test Kit.

### Peptide‐Centric Local Stability Assay

The cell membrane proteins of HGC27 cells were extracted using a cell membrane protein extraction kit. The extracted proteins were solubilized in M‐PER lysis buffer and quantified to a concentration of 1 mg mL^−1^. Subsequently, 50 µL of the protein lysate was incubated with either DMSO or SkII at 25 °C for 30 min. The samples were then subjected to limited proteolysis at a 1:2 (w/w) trypsin to substrate ratio for 1 min, followed by termination of digestion through the addition of 165 µL of guanidinium chloride solution. The digested samples were reduced with TCEP, alkylated with CAA, and heated at 95 °C for 5 min. The reaction mixtures were filtered through 10 kDa molecular weight cutoff centrifugal filters, and the filtrates were acidified with trifluoroacetic acid, desalted, and analyzed as DIA samples using an Orbitrap Exploris 480 mass spectrometer coupled with a micro‐flow LC system. Finally, the DIA spectra were processed and analyzed using Spectronaut software.

### Drug Affinity Responsive Target Stability

The cells were collected and lysed on ice with M‐PER lysis buffer for 30 min. The protein concentration of the cell lysate was quantified using a Bradford Assay Kit, which yielded a concentration of 5 mg mL^−1^. DMSO (1 µL) or 100× stock solutions of varying concentrations of SkII were added to 99 µL of cell lysate and incubated at room temperature for 3 h. Following incubation, the samples were aliquoted into 20 µL portions. To each sample tube, 2 µL of pronase solution (at an appropriate predetermined pronase concentration, with a ratio of protease to protein of 1:500) was added, while 2 µL of buffer was added to the control group in place of pronase. The samples were incubated at room temperature for 30 min. To stop the proteolytic activity, a protease inhibitor solution was added to each sample, mixed, and then frozen for 10 min. Finally, 6 µL of 5× loading buffer was added for subsequent analysis of target protein expression via Western blot to confirm the binding of SLC1A4 to SkII.

### Cellular Thermal Shift Assay

SkII was incubated with the cells for 3 h (with an equal volume of DMSO as the control) on ice. The samples were then evenly distributed and heated for 3 min at different temperatures (40–70 °C). Next, the cells underwent 3 freeze‐thaw cycles in liquid nitrogen. After centrifugation, the supernatant was collected into a new tube, and an appropriate amount of 5× loading buffer was added. Subsequent procedures were performed according to the Western blot protocol.

### Surface Plasmon Resonance

The binding affinity between the small molecule SkII and the protein SLC1A4 was measured by SPR using a Biacore 8K instrument (GE Healthcare). A CM5 sensor chip was employed to immobilize the target protein onto the sensor surface via standard amine coupling reactions in PBS running buffer at 25 °C. A gradient of SkII concentrations was injected into the flow channels to assess binding affinity. The KD was calculated using the Biacore 8K evaluation software.

### JC‐1 Fluorescent Staining

The MMP was evaluated using a JC‐1 staining assay kit according to the manufacturer's protocol. Briefly, HGC27 cells were incubated with JC‐1 reagent for 20 min at 37 °C under 5% CO_2_. Following incubation, the cells were washed twice with JC‐1 staining buffer and immediately visualized under an inverted fluorescence microscope for image acquisition.

### Transfection

Complementary oligonucleotide sequences of siSLC1A4s (Table S3, Supporting Information) were designed and synthesized by Genoagent Co., Ltd. (Shanghai, China). The full‐length cDNA encoding SLC1A4 were inserted into GV492 vector. HGC27 cells were then transiently transfected with plasmids and siSLC1A4s using Lipofectamine 3000 (Invitrogen).

### RNA‐Seq

Total RNA was isolated from four individual cell samples per group using TRIzol reagent. Reverse transcription and library construction were performed by LC‐Bio Technology Co., Ltd. (Hangzhou, China) according to the manufacturer's instructions. Paired‐end 2 × 150 bp sequencing (PE150) was conducted on an Illumina NovaSeq 6000 platform. After the final transcriptome was generated, StringTie and Ballgown were used to estimate transcript expression levels and assess mRNA abundance by calculating fragments per kilobase of transcript per million mapped reads (FPKM) values. Differential gene expression analysis between groups was performed using DESeq2, and edgeR was used for comparisons between two samples. Genes with *p* < 0.05 and FC ≥ 2 were considered differentially expressed. These differentially expressed genes were then subjected to KEGG pathway analysis.

### LC‐MS Analysis and Isotope Tracing Metabolic Flux Analysis

For cell sample preparation, HGC27 cells were seeded at a density of 3 × 10^6^ cells in 15 cm^2^ culture dishes. The cultured cells were treated with different concentrations of SkII (0 and 16 µM) for 48 h. After the original culture medium was removed, the cells were digested with trypsin, counted, and resuspended in 1.5 mL centrifuge tubes. Then, 1 mL of a chilled solvent mixture (methanol: acetonitrile: water = 50:30:20) was added, and the mixture was sonicated for 10 min at 4 °C. The centrifuge tubes were stored at −80 °C overnight to facilitate cell lysis and protein precipitation. Afterward, the samples were centrifuged at 12 000 × g for 10 min at 4 °C, and the supernatant was transferred to a new centrifuge tube. The extracts were then dried using a vacuum freeze dryer at 4 °C. The dried extracts were resuspended in 60 µL of a methanol: water (2:98) mixture, followed by sonication for 10 min at 4 °C. After another centrifugation at 12 000 × g for 10 min at 4 °C, 45 µL of the supernatant was transferred to an autosampler vial. Additionally, quality control (QC) samples were prepared by mixing equal volumes of the supernatants from all samples and were injected periodically (every seven samples) for metabolomics analysis using the Orbitrap Exploris 120 system.

For sample preparation of tumor tissues, a specific amount of tumor tissue was weighed and mixed with ten times its volume of ultrapure water along with steel beads for grinding. The tissue was then homogenized using a tissue grinder. Next, 200 µL of the tissue homogenate was aspirated and mixed with 800 µL of chilled methanol: acetonitrile (50:30) mixture. All subsequent procedures followed the same method as for the cell sample preparation.

For the cell isotope tracing metabolomics flux analysis, HGC27 cells were seeded at a density of 3 × 10^6^ cells in 15 cm^2^ culture dishes. The cultured cells were treated with different concentrations of SkII (0 and 16 µM) for 48 h. The original culture medium was removed and replaced with isotope tracing medium (either glucose‐free RPMI 1640 medium supplemented with 11 mM ^13^C_6_‐D‐glucose or L‐serine‐free RPMI 1640 medium supplemented with 400 µM ^15^N,^13^C_3_‐L‐serine) and incubated for 8 h. The cells were then collected for metabolic flux analysis.

The samples (10 µL) were injected into an Orbitrap Exploris 120 system equipped with an HSS T3 chromatographic column for separation. The analysis was conducted in positive ion mode, using water containing 0.1% formic acid as mobile phase A and acetonitrile as mobile phase B. The flow rate was set to 0.3 mL min^−1^, with the column maintained at 40 °C. The elution gradient was as follows: 0–1 min, 2% B; 1–4.5 min, 2–60% B; 4.5–7 min, 60% B; 7–8 min, 60–100% B; 8–11.4 min, 100% B; 11.4–11.5 min, 100–2% B; 14 min, 2%. The electrospray ionization (ESI) conditions were set as follows: spray voltage at 3.5 kV, sheath gas at 50 arb, auxiliary gas at 10 arb, and ion transfer tube temperature at 300 °C, with collision energy set to SNCE 20–40–60%. The mass range detected was m/z 60 to 900. These settings were chosen because positive ion mode combined with a water/acetonitrile gradient provides high sensitivity for amino acid and metabolite detection. Metabolite identification was performed using MS‐DIAL, primarily based on metabolite retention time, MS1, and MS/MS similarity for metabolite annotation. AccuCor2 was employed for natural abundance correction of isotopic data in labeling experiments with ^13^C_6_‐D‐glucose and ^15^N,^13^C_3_‐L‐serine.^[^ ^38^ ^]^

### Histology, IHC and TUNEL Assay

The tissue samples were embedded in paraffin after fixation in 4% paraformaldehyde for 24 h and sectioned at a thickness of 4 µm. Prior to analysis, the sections were deparaffinized and rehydrated. For histological examination, the tissue sections were stained with H&E.

Tumor tissues from 160 GC patients and model mice were fixed in 10% formaldehyde and embedded in paraffin. The tissues were sectioned, deparaffinized, rehydrated through a series of alcohol solutions, and subjected to antigen detection. The sections were blocked with 10% goat serum at 37 °C for 30 min. The slides were then incubated overnight at 4 °C with the primary antibody, followed by a 30 min incubation with the secondary antibody. DAB was used for visualization of the stained sections.

Deparaffinized and rehydrated tumor sections were permeabilized with proteinase K for 30 min, washed with PBS, and treated with a TUNEL apoptosis detection kit according to the manufacturer's instructions. Apoptosis in the tumor tissues was assessed using fluorescence microscopy.

### Organoid Culture and Treatment

Tumor tissue samples from GC patients were transferred to cancer organoid basal medium (Cat# B213152, bioGenous) supplemented with antibiotics, and washed twice. Necrotic cells and nonepithelial components, such as muscle or adipose tissue, were meticulously removed using sterile forceps. The remaining tumor tissue was minced into fragments of ≈0.5–2 mm^3^ using sterile scissors and transferred into a 1.5 mL microcentrifuge tube. Tumor digestion was performed by adding tumor tissue digestion solution (Cat# K601003, bioGenous) at a volume 50 greater than times that of the minced tissue, and incubating at 37 °C with gentle shaking (50–100 rpm) for 20–90 min. The digested suspension was then rinsed with Anti‐Adherence Rinsing Kit (Cat# E238002, bioGenous) and gently pipetted to promote complete dissociation. After digestion, FBS was added to inactivate the digestion enzymes, and the suspension was filtered through a 100 µm cell strainer. The filtered suspension was centrifuged at 200–300 x g for 3–5 min, and the supernatant was discarded. The remaining pellet was washed twice with cancer organoid basal medium. Cells were mixed with organoid culture ECM (Cat# M315066, bioGenous) at a ratio of 8000–30 000 cells per well to 20–30 µL ECM. The mixture was kept on ice and transferred into a 24‐well plate. After the ECM solidified, complete medium was gently added along the well wall, and the medium was changed every 3 days. To assess the effect of SkII on the organoids, the cultured organoids were treated with SkII at concentrations of 0, 4, 8, and 16 µM for 48 h. Images were captured using an Olympus CKX53 inverted fluorescence microscope. After SkII treatment, the organoids were fixed in 4% paraformaldehyde at room temperature for 30 min and washed with PBS. The organoids were then embedded and processed for IHC staining.

### Statistical Analysis

GraphPad Prism 10 software was used for graphing and statistical analysis, with all the error bars representing the means ± SDs. Detailed information regarding statistical analyses can be found in the figure legends. Flow cytometry data were analyzed using FlowJo (v10). Illustrations were created using BioRender. Metabolic pathway enrichment analysis was performed using the MetaboAnalyst platform (http://www.metaboanalyst.ca).^[^ ^39^ ^]^ The chi‐square test and Fisher's exact test were used to compare the categorical variables. The Mann‐Whitney U test was used to compare SLC1A4 expression levels between GC tissues and paracancerous tissues. The Kaplan‐Meier technique and the log‐rank test were used to determine the survival rates.

### Ethics Approval Statement

The study was reviewed and approved by the Ethics Committee of Zhejiang Cancer Hospital (Ethical ID: IRB‐2021‐431) and was conducted in accordance with the ethical principles outlined in the Declaration of Helsinki (2013 revision). Written informed consent was obtained from all participants and/or their legal guardians prior to their inclusion in the study.

## Conflict of Interest

The authors declare no conflict of interest.

## Author Contributions

J.Z. and Y.M. contributed equally to this work and co‐first authors. J.Z. performed conceptualization, data curation, formal analysis, methodology, validation, visualization, wrote the original draft, and reviewed and edited the final manuscript. Y.M. performed data curation, visualization, and wrote the original draft. R.X., Z.J., Y.Z., Y.W., M.Y., and J.D. assisted with the establishment of tumor mouse models. T.Z. performed funding acquisition, supervision, wrote the original draft, and reviewed and edited the final manuscript. L.B. and L.Y. performed funding acquisition, project administration, resources, supervision, wrote the original draft, and reviewed and edited the final manuscript.

## Supporting information



Supporting Information

## Data Availability

The data that support the findings of this study are available from the corresponding author upon reasonable request.;
