# Supplementary material for: Skullcapflavone II Inhibits SLC1A4‐Mediated L‐Serine Uptake and Promotes Mitochondrial Damage in Gastric Cancer
Source: Adv Sci (Weinh). 2025 Sep 19;12(45):e17225. doi: 10.1002/advs.202417225 (PMC12677694; doi:10.1002/advs.202417225)
Supplement: Supplementary file 1 — Supporting Information [file ADVS-12-e17225-s001.docx]

Supporting Information

**Table S1. Association between SLC1A4 expression and gastric cancer clinicopathological characteristics**

| Variables | Expression | | Total | High rate | χ2 | *P* value |
| --- | --- | --- | --- | --- | --- | --- |
|  | High | Low |  |  |  |  |
| Age (years) | | | | | | |
| >65 | 35 | 65 | 100 | 35.0% | 0.042 | 0.837 |
| ≤65 | 40 | 70 | 110 | 36.4% |  |  |
| Sex | | | | | | |
| Female | 22 | 35 | 57 | 38.6% | 0.283 | 0.595 |
| Male | 53 | 100 | 153 | 34.6% |  |  |
| Smoking | | | | | | |
| Yes | 17 | 34 | 51 | 33.3% | 0.148 | 0.700 |
| No | 57 | 100 | 157 | 36.3% |  |  |
| Drinking | | | | | | |
| Yes | 13 | 25 | 38 | 34.2% | 0.029 | 0.865 |
| No | 61 | 110 | 171 | 35.7% |  |  |
| Family history | | | | | | |
| Yes | 3 | 13 | 16 | 18.8% | 2.102 | 0.147 |
| No | 71 | 122 | 132 | 36.8% |  |  |
| Tumor location | | | | | | |
| Antrum | 26 | 64 | 90 | 28.9% | 3.564 | 0.313 |
| Body | 21 | 26 | 47 | 44.7% |  |  |
| Cardia fundus | 24 | 40 | 64 | 37.5% |  |  |
| Entire stomach | 1 | 2 | 3 | 33.3% |  |  |
| Grade of differentiation | | | | | | |
| Poor | 39 | 68 | 107 | 36.4% | 0.835 | 0.659 |
| Moderate-poor | 16 | 34 | 50 | 32.0% |  |  |
| Moderate or Well | 10 | 25 | 35 | 28.6% |  |  |
| Tumor size (cm) | | | | | | |
| ≥5 cm | 44 | 61 | 105 | 41.9% | 3.916 | **0.048** |
| <5 cm | 29 | 72 | 101 | 28.7% |  |  |
| TNM stage | | | | | | |
| I | 2 | 11 | 13 | 15.4% | 7.864 | **0.049** |
| II | 7 | 25 | 32 | 21.9% |  |  |
| III | 57 | 82 | 139 | 41.0% |  |  |
| IV | 8 | 8 | 16 | 50.0% |  |  |

**Table S2. The composition of the control and serine-glycine deficient (SG-) diet**

| Ingredient | Control diet | | Serine-glycine deficient (SG-) diet | |
| --- | --- | --- | --- | --- |
|  | gm | kcal | gm | kcal |
| L-Arginine-HCL | 16.00 | 64.00 | 16.00 | 64.00 |
| L-Histidine-HCL | 8.00 | 32.00 | 8.00 | 32.00 |
| L-Lysine HCl | 18.70 | 74.80 | 18.70 | 74.80 |
| L-Tyrosine | 5.30 | 21.20 | 5.30 | 21.20 |
| L-Tryptophan | 2.70 | 10.80 | 2.70 | 10.80 |
| L-Phenylalanine | 10.70 | 42.80 | 10.70 | 42.80 |
| L-Methionine | 8.00 | 32.00 | 8.00 | 32.00 |
| L-Cystine | 6.40 | 25.60 | 6.40 | 25.60 |
| L-Threonine | 10.70 | 42.80 | 10.70 | 42.80 |
| L-Leucine | 16.00 | 64.00 | 16.00 | 64.00 |
| L-Isoleucine | 10.70 | 42.80 | 10.70 | 42.80 |
| L-Valine | 10.70 | 42.80 | 10.70 | 42.80 |
| Glycine | 13.30 | 53.20 | 0.00 | 0.00 |
| L-Proline | 3.50 | 14.00 | 3.50 | 14.00 |
| L-Glutamine | 16.00 | 64.00 | 16.00 | 64.00 |
| L-Alanine | 3.50 | 14.00 | 3.50 | 14.00 |
| L-Aspartic Acid | 9.50 | 38.00 | 9.50 | 38.00 |
| L-Serine | 13.30 | 53.20 | 0.00 | 0.00 |
| Total L-Amino Acid | 183.00 | 732.00 | 156.40 | 625.60 |
| Cornstarch | 397.09 | 1588.00 | 423.64 | 1695.00 |
| Maltodextrin | 145.00 | 580.00 | 145.00 | 580.00 |
| Sucrose | 100.00 | 400.00 | 100.00 | 400.00 |
| Cellulose | 50.00 | 0.00 | 50.00 | 0.00 |
| Soybean Oil | 70.00 | 630.00 | 70.00 | 630.00 |
| Choline Chloride | 2.50 | 0.00 | 2.50 | 0.00 |
| TBHQ | 0.01 | 0.00 | 0.01 | 0.00 |
| Mineral Mix S10022G | 35.00 | 0.00 | 35.00 | 0.00 |
| Vitamin Mix V10037 | 10.00 | 0.00 | 10.00 | 0.00 |
| Sodium Bicarbonate | 7.40 | 40.00 | 7.40 | 40.00 |
| FD&C Blue Dye | 0.00 | 0.00 | 0.05 | 0.30 |
| Total | 1000.00 | 3970.00 | 1000.00 | 3970.90 |

**Table S3. Primer and siRNA sequence**

| Primer | |
| --- | --- |
| SLC1A4 F | TGGTCCTGTTTGCTCTGGTG |
| SLC1A4 R | AGGGTCGCTGAGCACATAAT |
| PSPH F | GCGATGGGAGTTGAGACGTA |
| PSPH R | CAAGAGCAAGAGCCCTGTGT |
| GAPDH F | TCCAAAATCAAGTGGGGCGA |
| GAPDH R | TGATGACCCTTTTGGCTCCC |
| siRNA | |
| si-SLC1A4 #1 F | CCUGUUUGCUCUGGUGUUAdTdT |
| si-SLC1A4 #1 R | UAACACCAGAGCAAACAGGdTdT |
| si-SLC1A4 #2 F | CGCGGUGUUCAUUGCGCAAdTdT |
| si-SLC1A4 #2 R | UUGCGCAAUGAACACCGCGdTdT |
| si-SLC1A4 #3 F | GGUGUGGACAAGAGGAUCAdTdT |
| si-SLC1A4 #3 R | UGAUCCUCUUGUCCACACCdTdT |


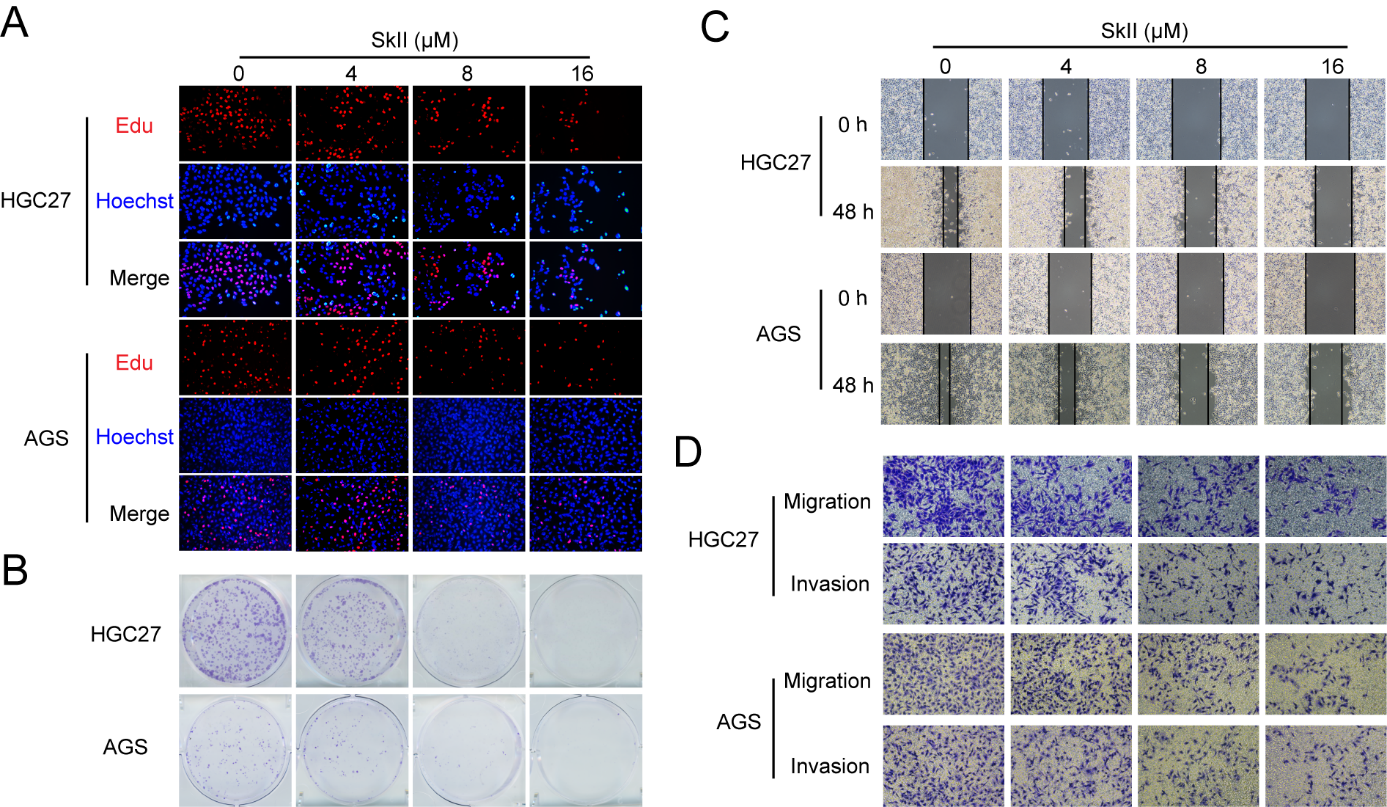


**Figure S1. SkII inhibits the proliferation, invasion, and migration of gastric cancer cells in vitro.**

A) Representative images of EdU assay showing the effect of SkII (0–16 μM) on the proliferation of HGC27 and AGS cells after 48 hours of treatment.

B) Representative images of the colony formation assay showing the effect of SkII (0–16 μM) on HGC27 and AGS cells after 48 hours of treatment.

C) Representative images of the wound healing assay showing the effect of SkII (0–16 μM) on the migration of HGC27 and AGS cells after 48 hours of treatment.

D) Representative images of the Transwell invasion and migration assays showing the effect of SkII (0–16 μM) on HGC27 and AGS cells after 48 hours of treatment.


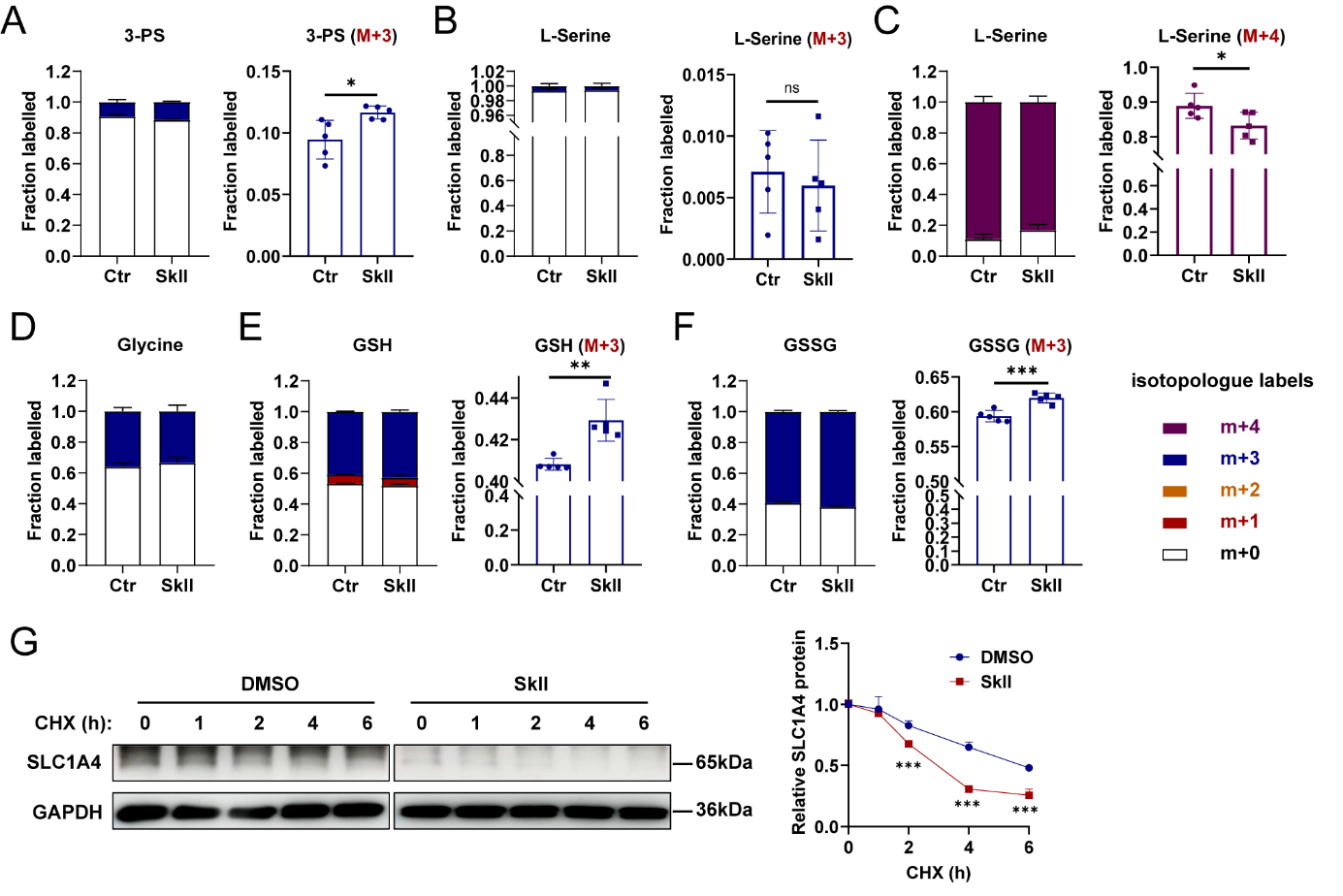


**Figure S2. Isotope-labeled fractions of ^13^C_6_-D-glucose and ^15^N,^13^C_3_-L-serine under SkII treatment and SKII accelerates SLC1A4 degradation.**

A) ^13^C_6_-glucose tracing into 3-PS.

B) ^13^C_6_-glucose tracing into L-serine.

C) ^15^N,^13^C_3_-L-serine tracing into L-serine.

D) ^15^N,^13^C_3_-L-serine tracing into glycine.

E) ^15^N,^13^C_3_-L-serine tracing into GSH.

F) ^15^N,^13^C_3_-L-serine tracing into GSSG.

G) Western blot analysis of SLC1A4 protein levels following treatment with 40 μg/mL cycloheximide (CHX) for 0, 1, 2, 4 and 6h after pretreatment with DMSO or SkII (16 μM) for 48h. GAPDH was used as the loading control. In all statistical graphs, data are presented as mean ± SD, and statistical significance was determined by one-way ANOVA or unpaired Student's t-test (ns, not significant; *p < 0.05, **p < 0.01, ***p < 0.001).


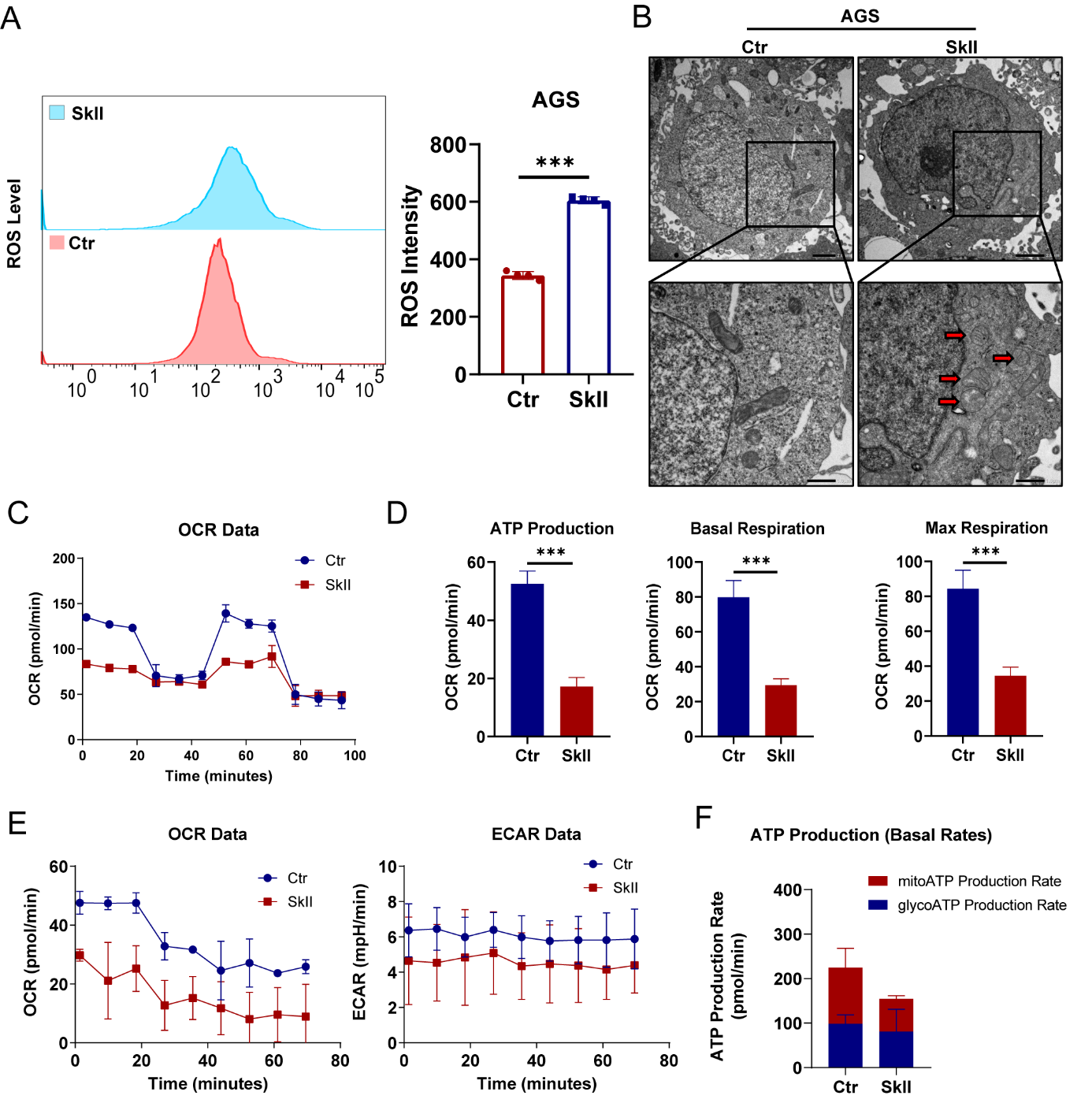


**Figure S3. SkII increases mitochondrial ROS accumulation in AGS cells, promoting mitochondrial damage and inhibiting mitochondrial energy metabolism.**

A) Flow cytometry analysis of ROS levels in AGS cells treated with SkII (0 and 16 μM) for 48 hours.

B) TEM analysis of mitochondrial structure in AGS cells treated with SkII (0 and 16 μM) for 48 hours.

C) Mitochondrial stress test in AGS cells treated with SkII (0 and 16 μM) for 48 hours.

D) OCR analysis of ATP production, basal respiration, and maximal respiration in AGS cells treated with SkII (0 and 16 μM) for 48 hours.

E) Real-time ATP rate assay in AGS cells treated with SkII (0 and 16 μM) for 48 hours.

F) ATP production rate analysis in AGS cells treated with SkII (0 and 16 μM) for 48 hours. In all statistical graphs, data are presented as mean ± SD, and statistical significance was determined by one-way ANOVA or unpaired Student's t-test (ns, not significant; *p < 0.05, **p < 0.01, ***p < 0.001).


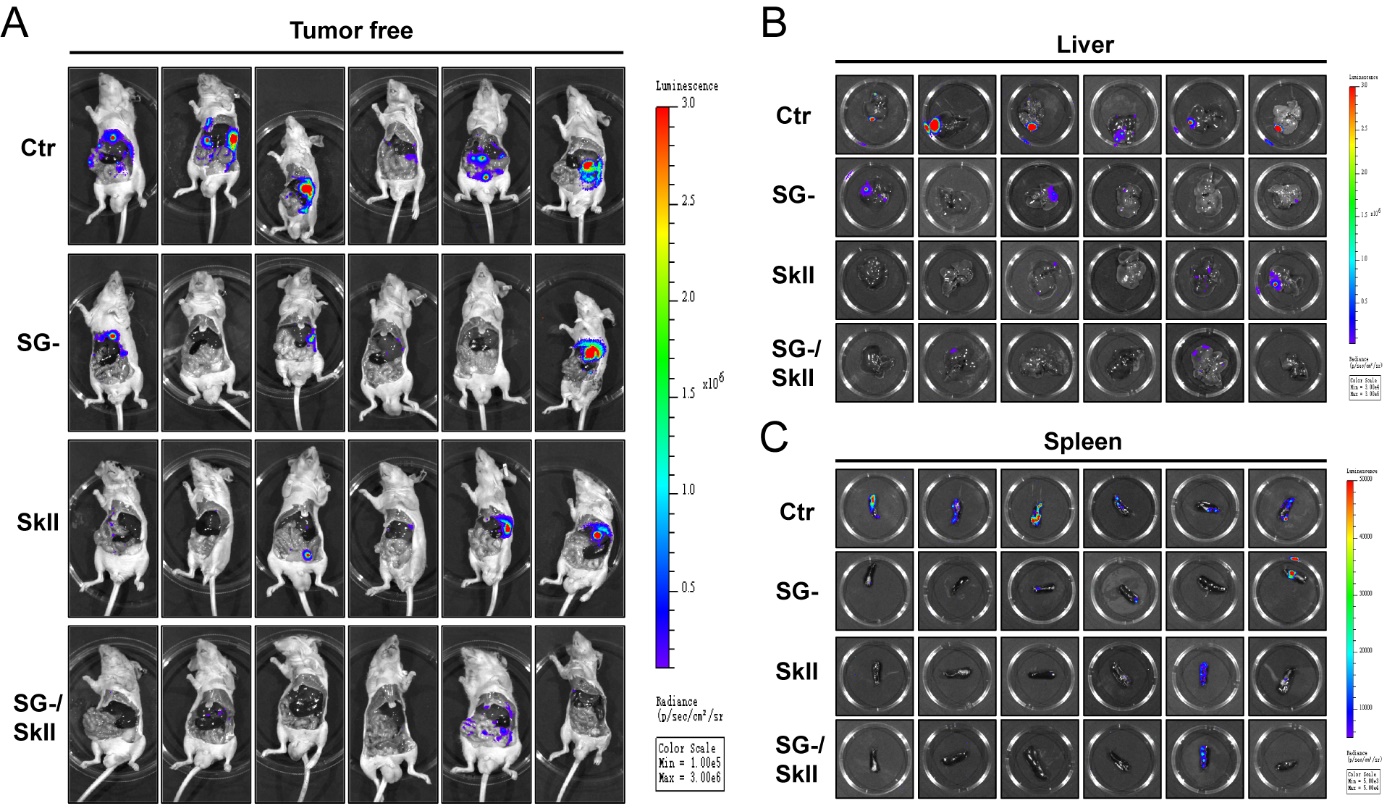


**Figure S4. SkII inhibits tumor metastasis in the abdominal cavity, liver, and spleen.**

A) Fluorescence images of abdominal metastases in mice after 30 days of serine/glycine-restricted diet or SkII treatment (n = 6).

B) Fluorescence images of liver metastases in mice after 30 days of serine/glycine-restricted diet or SkII treatment (n = 6).

C) Fluorescence images of spleen metastases in mice after 30 days of serine/glycine-restricted diet or SkII treatment (n = 6).


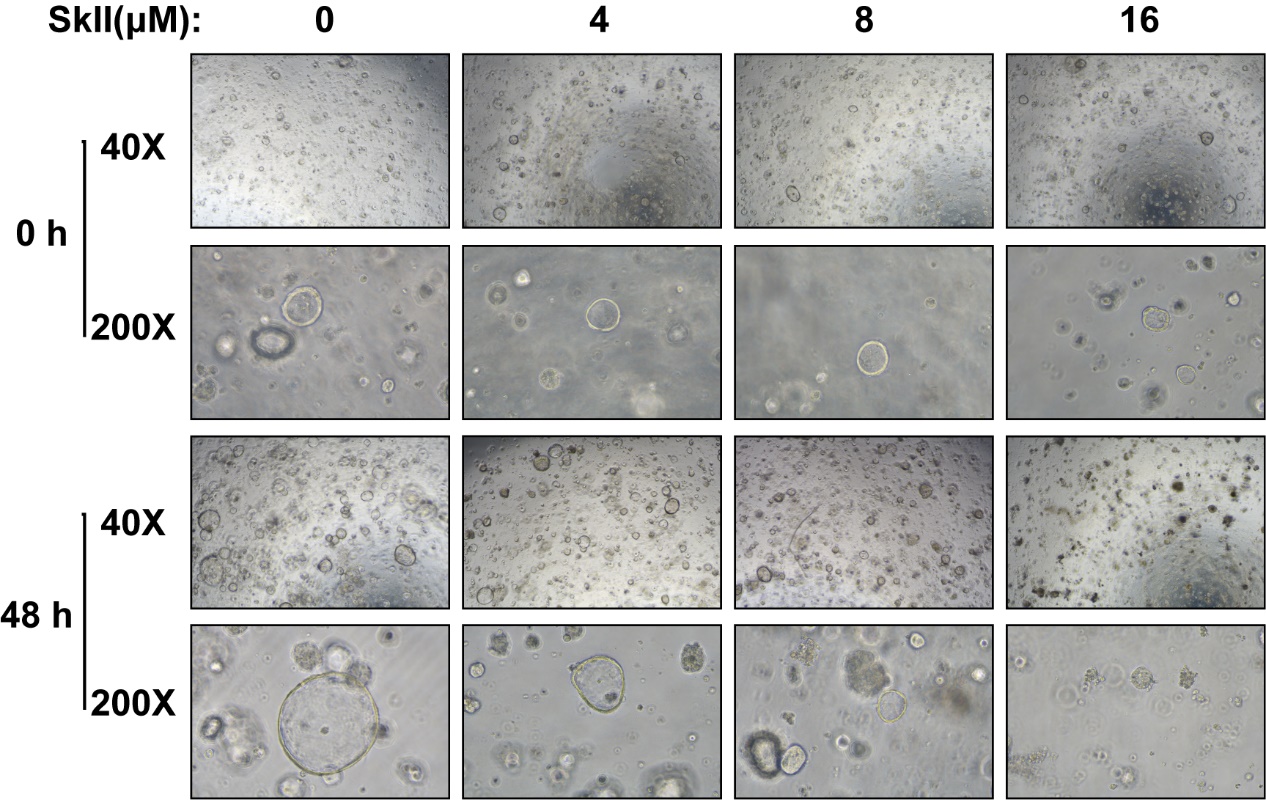


**Figure S5. Representative images of gastric cancer organoids treated with SkII (0–16 μM) for 48 hours.**
